# CNS-dominant human FMRP isoform rescues seizures, fear, and sleep abnormalities in *Fmr1*-KO mice

**DOI:** 10.1172/jci.insight.169650

**Published:** 2023-06-08

**Authors:** Hayes Wong, Alexander W.M. Hooper, Hye Ri Kang, Shiron J. Lee, Jiayi Zhao, Chanchal Sadhu, Satinder Rawat, Steven J. Gray, David R. Hampson

**Affiliations:** 1 Department of Pharmaceutical Sciences, University of Toronto, Toronto, Ontario, Canada.; 2 University of Texas Southwestern Medical Center, Dallas, Texas, USA.; 3 Taysha Gene Therapies Inc., Dallas, Texas, USA.; 4 Department of Pharmacology and Toxicology, University of Toronto, Toronto, Ontario, Canada.

**Keywords:** Neuroscience, Therapeutics, Gene therapy, Mental retardation, Neurodevelopment

## Abstract

Fragile X syndrome is a neurodevelopmental disorder caused by the absence of the mRNA-binding protein fragile X messenger ribonucleoprotein (FMRP). Because FMRP is a highly pleiotropic protein controlling the expression of hundreds of genes, viral vector–mediated gene replacement therapy is viewed as a potential viable treatment to correct the fundamental underlying molecular pathology inherent in the disorder. Here, we studied the safety profile and therapeutic effects of a clinically relevant dose of a self-complementary adeno-associated viral (AAV) vector containing a major human brain isoform of FMRP after intrathecal injection into wild-type and fragile X–KO mice. Analysis of the cellular transduction in the brain indicated primarily neuronal transduction with relatively sparse glial expression, similar to endogenous FMRP expression in untreated wild-type mice. AAV vector–treated KO mice showed recovery from epileptic seizures, normalization of fear conditioning, reversal of slow-wave deficits as measured via electroencephalographic recordings, and restoration of abnormal circadian motor activity and sleep. Further assessment of vector efficacy by tracking and analyzing individual responses demonstrated correlations between the level and distribution of brain transduction and drug response. These preclinical findings further demonstrate the validity of AAV vector–mediated gene therapy for treating the most common genetic cause of cognitive impairment and autism in children.

## Graphical abstract

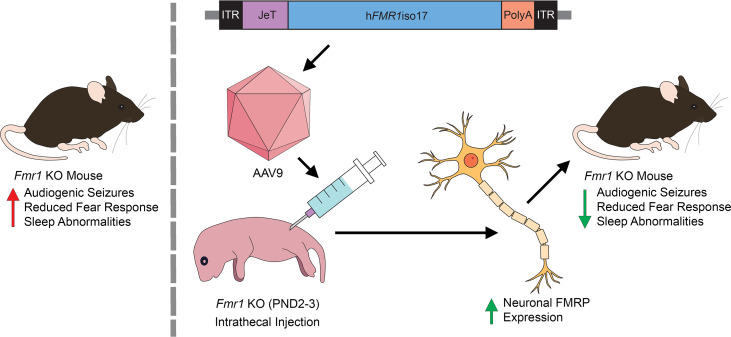

## Introduction

Neurodevelopmental disorders associated with cognitive impairment and autism have numerous known genetic causes. Among these, fragile X syndrome (FXS) is the most common single-gene cause of these pathological attributes. As with almost all other neurodevelopmental disorders, therapy for FXS has been restricted to the administration of small molecule drugs to suppress symptoms that include hyperactivity, anxiety, irritability, aggression, depression, and epileptic seizures. The genetic cause of FXS, a triplet repeat expansion in the 5′ noncoding region of the *FMR1* gene, results in the absence of or drastic reduction in the expression of the encoded protein, fragile X messenger ribonucleoprotein (FMRP, formerly known as fragile X mental retardation protein). Most organs, including the brain and gonadal organs, express FMRP. FMRP is an mRNA-binding protein that regulates gene expression and also acts as an essential intracellular mediator of the trafficking of axonal and synaptic proteins. Thus, because the disorder is caused by deficient FMRP, and because of FMRP’s pleiotropic nature, viral vector–mediated gene replacement therapy is increasingly viewed as a promising long-term and more comprehensive treatment.

Several studies have demonstrated the efficacy of adeno-associated viral (AAV) vector–mediated expression of FMRP in the mouse and rat knockout models of FXS. An early study reported normalization of long-term synaptic depression after injection of AAV5-FMRP into the hippocampus (1). Subsequent studies utilized neonatal intracerebroventricular injections of AAV9 vectors encoding mouse, rat, and human homologs of FMRP (2–5). In these cases, a wide variety of abnormal behaviors were fully or partially corrected by AAV9-FMRP treatment. A complicating factor for gene replacement therapy is the presence of multiple *FMR1* alternatively spliced isoforms; at least 15 distinct mRNA isoforms have been reported (6–9). Because of limitations on the length of DNA that can be carried by AAV vectors, and the length of the *FMR1* DNA-coding region, only a single *FMR1* isoform can be packaged into a single AAV vector. Thus, the choice of the *FMR1* isoform, along with other parameters such as the route of administration, the gene promoter element, and the dose, all contribute critically to therapeutic efficacy.

The goal of the present study was to design and test a gene therapy protocol in a mouse model of FXS that more closely mimics a human therapeutic situation. We used intrathecal (i.t.) injections instead of the intraventricular injections used previously (2, 4), because this route of AAV administration is the most commonly used route for intra–cerebrospinal fluid (intra-CSF) injections in patients. A self-complementary AAV vector encoding an abundant human FMRP homolog (isoform 17) was used rather than single-stranded AAV vectors used in previous studies of FXS (1–5), because self-complementary AAV vectors mediate faster and more efficient vector transduction. Thus, self-complementary AAV vectors are more optimally suited for shorter therapeutic windows and may allow the use of lower doses compared with single-stranded vectors (10, 11). Additionally, clinically applicable efficacy-testing procedures, such as electroencephalogram (EEG) recordings and analysis of sleep and circadian rhythms, were employed (12–14). Importantly, because of high mouse-to-mouse variability in terms of the success of injection, transgene expression, and behavioral outcomes, we tracked individual mice and were able to demonstrate correlations between the level of FMRP expression and normalization of behavior and brain physiology.

## Results

### Expression of the scAAV-JeT-hFMR1iso17 vector.

**Figure 1:**
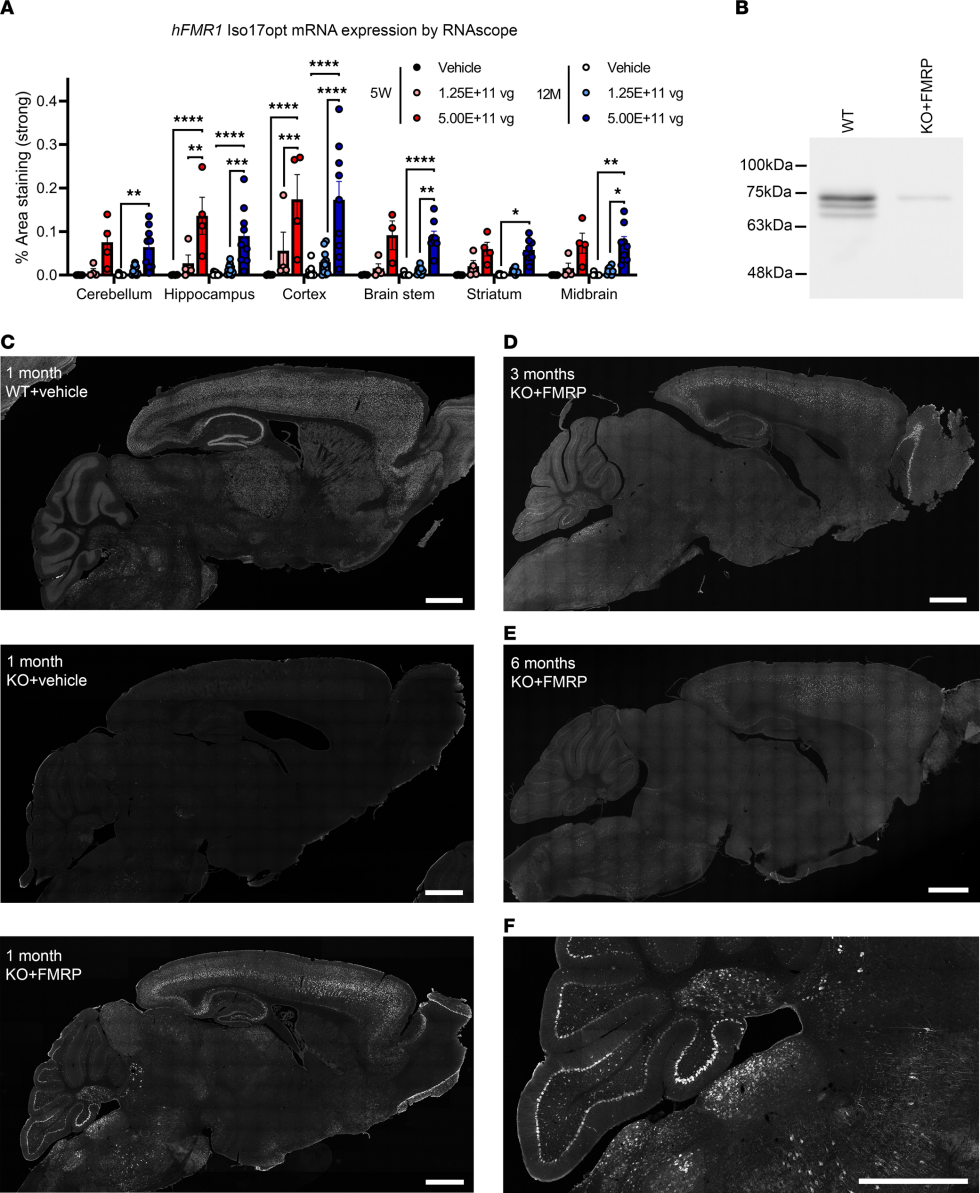
CNS expression profile of scAAV-JeT-h*FMR1*iso17 vector after intrathecal injection. (**A**) h*FMR1* Iso17 codon-optimized (opt) mRNA expression in the brain. Brains were collected and analyzed at 5 weeks and 12 months. RNAscope was performed with specific probes for h*FMR1* Iso17 opt mRNA. Histology images were analyzed using custom analysis settings in the HALO image analysis platform to quantify the signals. Two-way ANOVA, *P < 0.05, **P < 0.005, ***P < 0.001, ****P < 0.0001. Data are shown as mean ± SEM. (**B**) The relative molecular weight of FMRP expressed from the scAAV-JeT-h*FMR1*iso17 vector comigrates with the most highly expressed FMRP isoform(s) in the brainstem of the 1-month-old mouse. (**C**–**F**) Representative images of FMRP expression in brains of WT mice injected with vehicle and *Fmr1*-KO mice injected with either vehicle or the scAAV-JeT-h*FMR1*iso17 vector at PND 2–3, then collected at 1 month (**C**, top, middle, and lower panels correspond to WT+vehicle, KO+vehicle, and KO+FMRP), 3 months (**D**), or 6 months (**E**) postinjection. Expression of FMRP from the scAAV-JeT-h*FMR1*iso17 vector was present in the cerebral cortex, hippocampus, inferior and superior colliculus, thalamus, cerebellum, and brainstem. Scale bars = 1 mm. (**F**) Higher magnification image of KO+FMRP brain in **C**, showing extensive staining in the Purkinje and granule cell layers of the cerebellum and in the brainstem. Scale bar = 1 mm.

A total of 345 wild-type (WT) and *Fmr1*-knockout (*Fmr1*-KO) mice were injected with AAV-JeT-h*FMR1*iso17 vector or vehicle for this study. Except for the RNAscope and safety-testing experiments, all other mice in the AAV-FMRP treatment group received a dose of 2.3 × 10^11^ vector genomes (vg) per mouse. To verify the AAV-JeT-h*FMR1*iso17 vector (AAV-FMRP) was transcribed in the mouse CNS, RNAscope analysis was performed on samples dissected from CNS regions of WT mice at both 5 weeks and 12 months of age, after i.t. injections at postnatal day (PND) 7–10. In this experiment, 2 doses were studied: a low dose (1.3 × 10^11^ vg) and a high dose (5.0 × 10^11^ vg) of the vector (Figure 1A). Specific mRNA transcription of the human codon-optimized FMRP isoform 17 transgene was present in the brainstem, cerebellum, midbrain, striatum, hippocampus, and cerebral cortex at 5 weeks and at 12 months of age. Transcription of the transgene was significantly higher in the high-dose group compared with the low-dose group, particularly in the hippocampus, cerebral cortex, midbrain, and brainstem, signifying a dose-response relationship with the vector.

Our decision to use human isoform 17 of FMRP in this vector was based on isoform 17 and its rodent orthologs being the one of the most abundantly expressed isoforms of FMRP in the human, mouse, and rat CNS. To verify that the AAV-JeT-h*FMR1*iso17 vector was expressing the desired isoform, Western blotting was performed to compare the endogenous FMRP expressed in the WT mouse against the gene product of the AAV vector from an *Fmr1*-KO mouse. The AAV vector–generated FMRP transgene comigrated with the mostly highly expressed FMRP isoform in the WT mouse brain (Figure 1B). Of note, while most of the gene sequence was codon-optimized, we deliberately left the original G-quartet motif sequence unaltered; this was to allow the codon-optimized *FMR1* isoform 17 mRNA to interact with the FMRP RGG domain, to maintain the function of posttranscriptional control of the transgene mRNA (15, 16).

FMRP is endogenously expressed throughout the CNS at all ages; therefore, we sought to obtain as widespread expression in the brain as possible. To examine the distribution of the FMRP transgene in mice injected at PND 2 or 3, immunofluorescence staining of the CNS in mice treated with AAV-FMRP was performed in mice collected at 1 month (Figure 1, C and F), 3 months (Figure 1D) and 6 months (Figure 1E) postinjection, which corresponded with the testing ages at behavioral and safety testing. Expression of FMRP was observed in the cerebral cortex, hippocampus, inferior and superior colliculus, thalamus, Purkinje and granule cell layers of the cerebellum, and brainstem of KO+FMRP mice 1 month, 3 months, and 6 months postinjection (Figure 1, C–F). Transgene FMRP was present in the cerebral cortical layers II, III, V, and VI, with layers II/III and V showing the highest expression (Supplemental Figure 1; supplemental material available online with this article; https://doi.org/10.1172/jci.insight.169650DS1).

No negative behavioral, serological, or histopathological outcomes were observed after injection of AAV-FMRP into WT mice (injected at PND 7–10, behavior tested at 3 months postinjection, serum collected at 5 weeks and 12 months of age) or *Fmr1*-KO mice (injected at PND 2, serum and tissue samples collected at 6 months of age; Supplemental Figures 2 and 3).

### Cell type specificity and coverage of FMRP.

**Figure 2:**
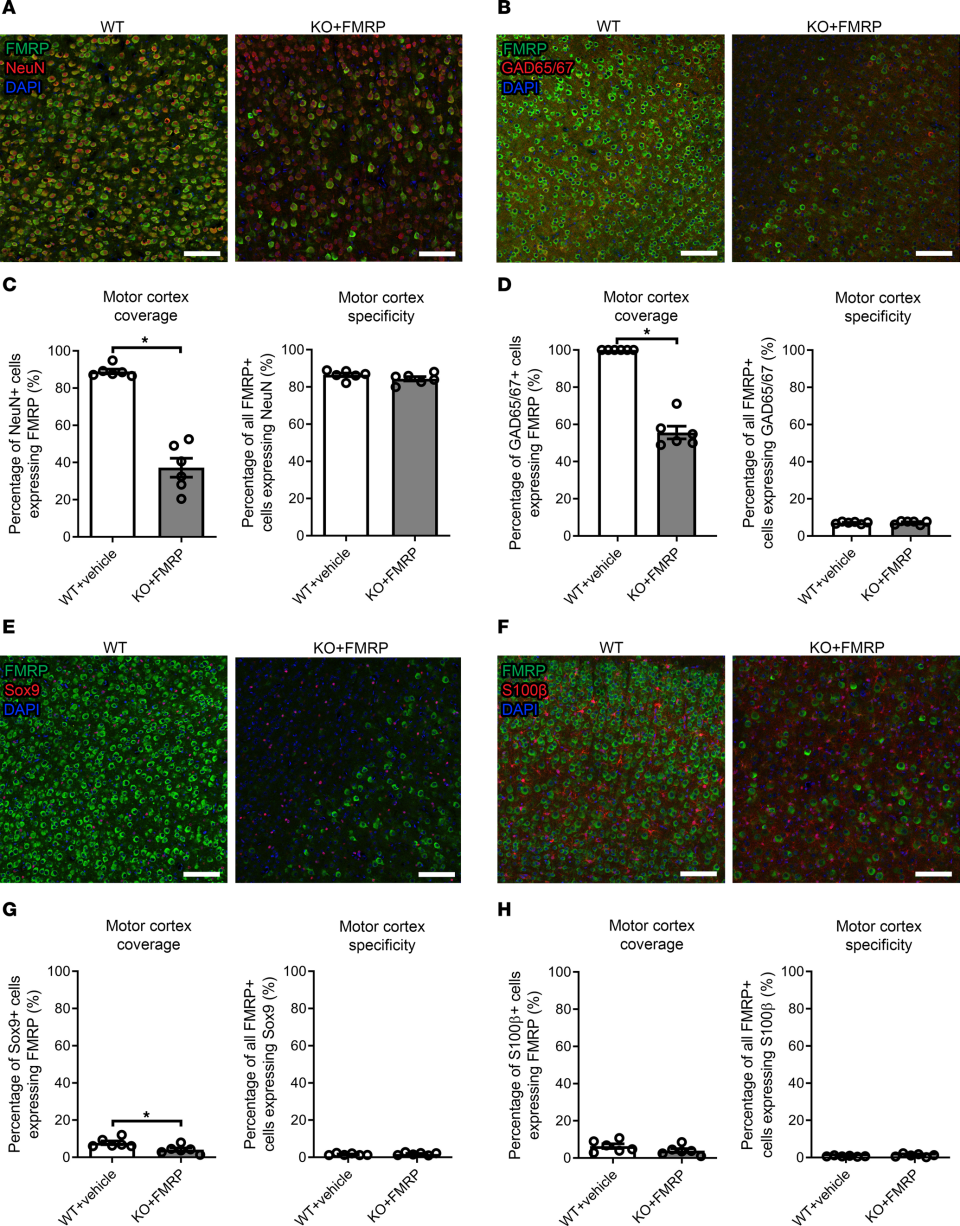
Cell type expression of scAAV-JeT-h*FMR1*iso17 vector. The motor cortices of WT+vehicle and KO+FMRP mice were immunolabeled and quantified at 2 weeks of age for colocalization of FMRP and NeuN (**A** and **C**, all neurons), glutamate decarboxylase 65/67 (GAD65/67) (**B** and **D**, GABAergic inhibitory neurons), Sox9 (**E** and **G**, astrocytes), or S100β (**F** and **H**, astrocytes, oligodendrocytes, oligodendroglial progenitor cells). Scale bars = 100 μm. The cell type specificity of FMRP from the scAAV-JeT-h*FMR1*iso17 vector is similar to endogenous FMRP expression in WT+vehicle mice. Coverage of cell types with FMRP from the scAAV-JeT-h*FMR1*iso17 vector is significantly lower than the endogenous FMRP in WT+vehicle mice for NeuN-, GAD65/67-, and Sox9-positive cells and is likely due to physical distribution of the AAV vector. *n*-values, 6 mice for each group. *P < 0.05, Student’s *t* test. Bars = mean ± SEM.

To reproduce the WT expression profile of FMRP as closely as possible, we sought to generate an AAV vector that transduced as many neuronal populations as possible, while limiting expression in glia, as glial expression of FMRP is low in adult WT mice (17). Analysis of the cell type specificity (the percentage of FMRP^+^ cells expressing a given cell type marker) of the FMRP transgene in the motor cortex of 2-week-old KO+FMRP mice revealed a cell type specificity profile very similar to that of WT+vehicle mice, with a mean specificity of the vector for NeuN^+^ neurons of 84% and 86%, respectively (Figure 2, A and C). Both the WT+vehicle and KO+FMRP treatment groups had low FMRP specificity for GAD65/67^+^ GABAergic neurons (Figure 2, B and D), with mean specificities of approximately 7%. WT+vehicle and KO+FMRP mice also both showed low specificity of FMRP expression in astrocytes, with mean specificities for Sox9^+^ cells of approximately 2% in both cases (Figure 2, E and G). There was similar low specificity of the vector for S100β^+^ cells (a marker for astrocytes, oligodendrocytes, and oligodendroglial progenitor cells), with a mean specificity of 0.7% in WT+vehicle mice and 1.2% in KO+FMRP mice (Figure 2, F and H). The mean coverage (the percentage of cells of a given cell type expressing FMRP) of FMRP expression in NeuN^+^ cells was significantly lower in KO+FMRP cortices than in WT+vehicle mice (88% vs. 37%, *P* < 0.05, Figure 2C), as was coverage of GAD65/67 (100% vs. 56%, *P* < 0.05, Figure 2D) and Sox9 (7% vs. 4%, *P* < 0.05, Figure 2G), though in the case of Sox9, the effect size was very small. Based on the transduction pattern after i.t. AAV injection (Figure 1) compared with previous work with intracerebroventricular AAV-FMRP–injected mice, it is likely the difference in coverage of neurons, GABAergic neurons, and astrocytes between WT+vehicle and KO+FMRP is due to limitations of the movement of AAVs through the CNS (2, 4). No significant difference was found in the coverage of S100β^+^ cells between WT+vehicle and KO+FMRP mice (Figure 2H). Together these cell specificity results demonstrate that the vector design and administration approach are capable of producing a desirable cell type expression profile for FMRP in *Fmr1*-KO mice similar to that of WT mice.

### Audiogenic seizure testing.

**Figure 3:**
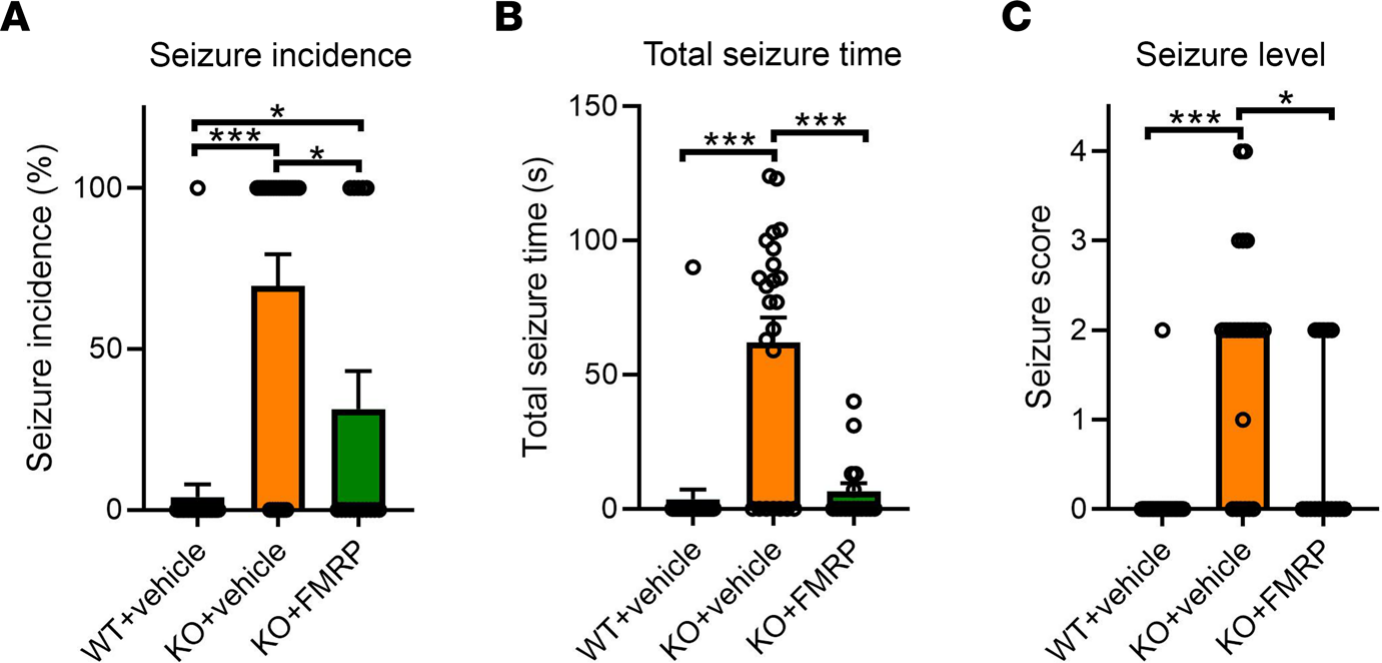
Rescue of audiogenic seizures in *Fmr1*-KO mice. (**A**) Both WT+vehicle and KO+FMRP mice had significantly lower incidence of seizure during the audiogenic seizure test, relative to KO+vehicle mice, although KO+FMRP mice showed higher incidence of seizures relative to WT+vehicle mice. Both WT+vehicle and KO+FMRP mice had significantly lower total seizure time (**B**) and seizure score (**C**) during audiogenic seizure test relative to KO+vehicle mice, indicating a rescue in seizure severity. *n* values: WT+vehicle = 25; KO+vehicle = 23; KO+FMRP = 16. *P < 0.05, ***P < 0.001. Seizure incidence: bars = mean ± SEM; Fisher’s exact test. Seizure time: bars = mean ± SEM, ANOVA, Tukey’s post hoc test. Seizure level: bars = median ± 95% CI, Kruskal-Wallis, Dunn’s post hoc test.

Increased susceptibility to audiogenic seizures (AGSs) is a robust endophenotype of *Fmr1*-KO mice (18, 19). To date, no gene therapy studies have reported rescue of this behavior in *Fmr1*-KO mice or rats. As expected, KO+vehicle mice had a greatly increased seizure incidence (Figure 3A), total seizure time (Figure 3B), and seizure level (Figure 3C), relative to WT+vehicle mice, with only 1 WT+vehicle mouse (4%) experiencing a seizure. Treatment with AAV-FMRP caused a striking reduction in seizure incidence (31%), mean total seizure time (7 seconds), and maximum seizure score (2 – clonic seizure) in *Fmr1*-KO mice, relative to KO+vehicle mice. We believe this is the first demonstration of rescue of AGSs in *Fmr1*-KO mice via gene therapy, and it illustrates the utility of this treatment in protection against CNS hyperactivity.

### Fear conditioning.

**Figure 4:**
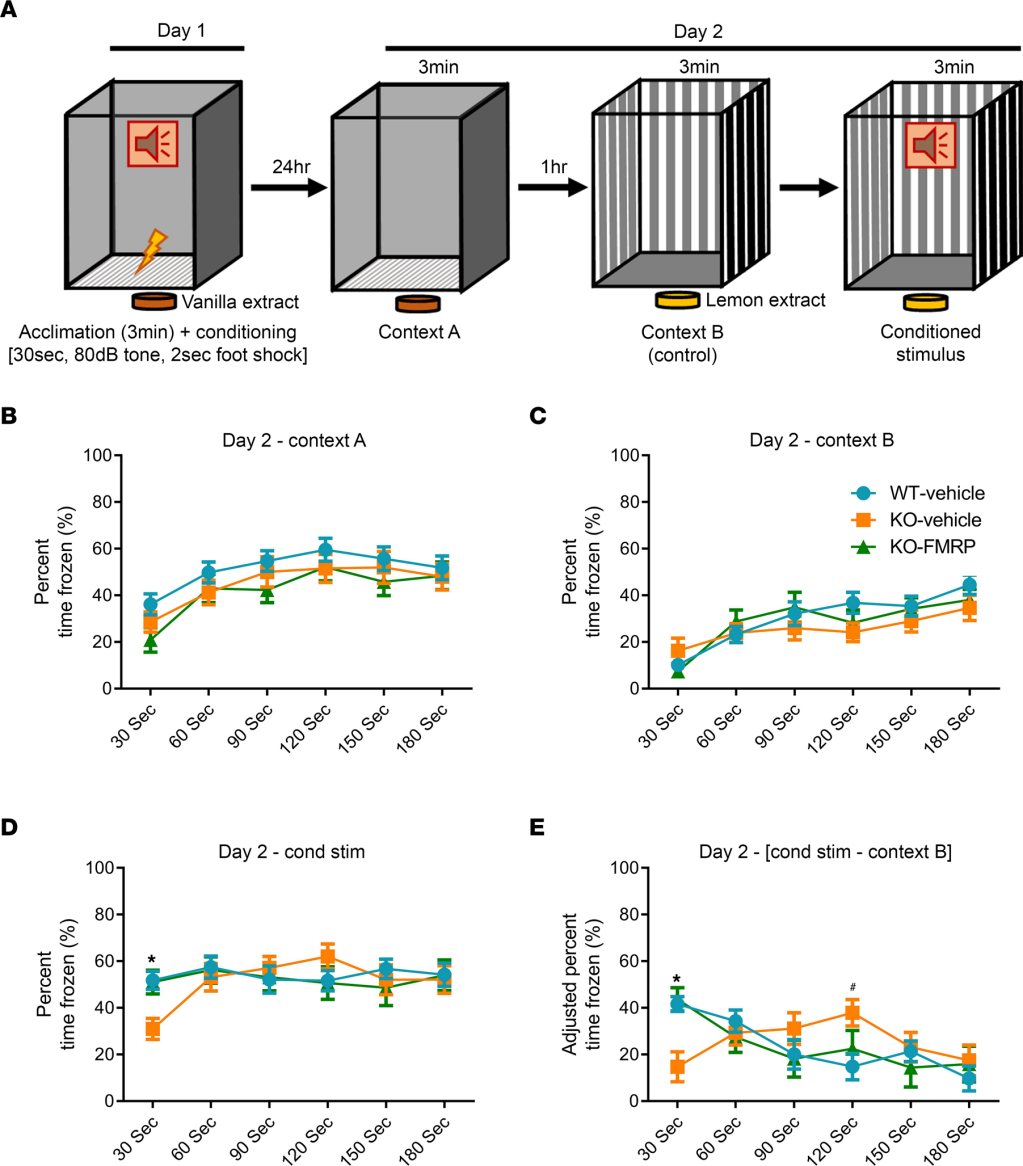
Normalization of fear memory response to conditioned stimulus in mice treated with scAAV-JeT-h*FMR1*iso17 vector. (**A**) Schematic of the fear conditioning protocol. (**B**–**E**) Results from the fear conditioning test. No differences were found among genotypes or treatments in response to the conditioned context (**B**, context A) or a novel context (**C**, context B). KO+vehicle mice froze significantly less than both WT+vehicle and KO+FMRP mice in the first 30 seconds of exposure to the conditioned stimulus (**D**). This effect remained after correcting for inherent freezing activity on a per-animal basis (cond stim — context B), and KO+vehicle mice also demonstrated a significantly higher rate in freezing relative to WT+FMRP mice during the 90–120 seconds interval of exposure to the conditioned tone (**E**). All mice were females. *n* values: WT+vehicle = 27; KO+vehicle = 19; KO+FMRP = 14. Bars = mean ± SEM. *P < 0.05 for 1-way ANOVA and Tukey’s post hoc test comparing percentage of time frozen between KO+vehicle mice and both WT+vehicle and KO+FMRP mice. ^#^*P* < 0.05 for 1-way ANOVA and Tukey’s post hoc test comparing percentage of time frozen between KO+vehicle mice and WT+vehicle mice.

In order to test fear memory behavior of KO+FMRP mice to contextual and conditioned stimulus cues, mice underwent a fear conditioning test (Figure 4A). In preliminary testing, no difference was observed in the behavior of male WT and KO mice in the fear conditioning test, and so only female mice were tested. There were no significant differences among WT+vehicle, KO+vehicle, or KO+FMRP mice in the freezing time when exposed to a fear-conditioned context (Figure 4B). There was also no difference in freezing time between WT+vehicle, KO+vehicle, or KO+FMRP mice, when placed into a novel context (Figure 4C). However, KO+vehicle mice froze significantly less than both WT+vehicle mice in the first 30 seconds of exposure to a fear-conditioned tone, indicating that KO+vehicle mice have a reduced fear memory to the conditioned stimulus, increased fearlessness, or an alternative nonfreezing behavioral response to fear and that FMRP is important to this endophenotype (Figure 4D). This altered freezing behavior was not present in KO+FMRP mice, indicating that AAV-FMRP treatment is sufficient to rescue this behavior. The reduced freezing in KO+vehicle mice and the rescue of freezing in KO+FMRP mice remained after correcting for the endogenous freezing rate of the mice on a per-mouse basis (Figure 4E). Interestingly, after this correction was made, it was noted that the percentage time frozen in KO+vehicle mice was significantly higher than WT+vehicle mice in the 90–120 seconds interval. This could indicate a delayed freezing response to the conditioned tone, though further experimentation would be required to verify this effect. While the fear conditioning test is a complex measurement of several behavioral characteristics, these results demonstrate that i.t. treatment with AAV-FMRP is capable of fully restoring these behaviors to WT levels.

### Circadian locomotor activity and sleep analyses.

**Figure 5:**
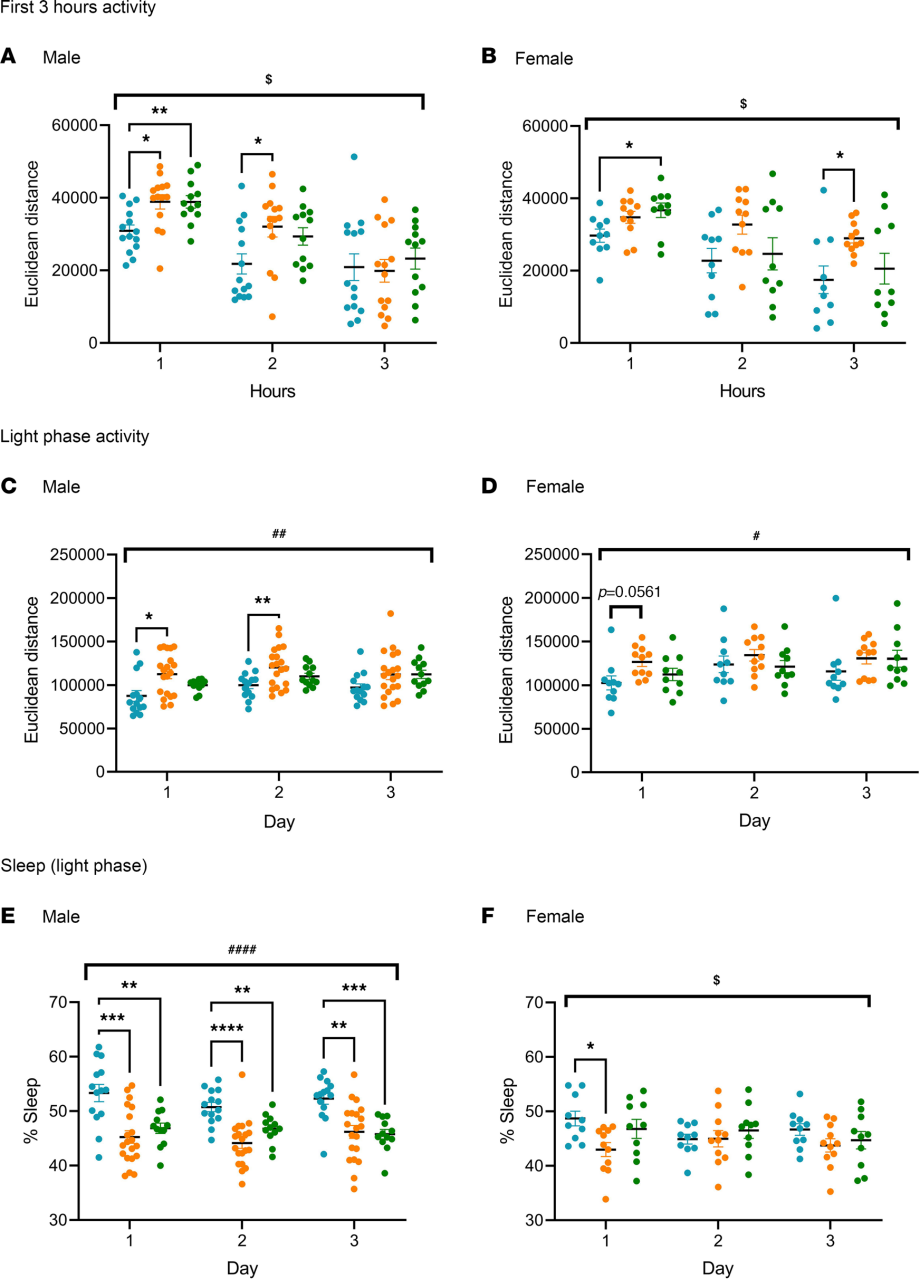
Effect of AAV-FMRP treatment on circadian locomotor activity of *Fmr1*-KO mice. (**A** and **B**) Locomotor activity in the initial 3 hours after being placed in the activity-recording apparatus. Blue dots = WT+vehicle, orange dots = KO+vehicle, green dots = KO+FMRP. The KO+vehicle group exhibited hyperactivity, which was reduced in the KO+FMRP groups: second hour in the male mice (**A**) and third hour in the female mice (**B**). (**C** and **D**) Locomotor activity in the light phase during 3 days of recording. In the male mice, AAV-FMRP treatment reduced hyperactivity observed in the first and second day compared with the KO+vehicle mice (**C**). In the female mice, a similar trend was observed in the first day, but the increase in activity in the KO+vehicle group was not statistically significant (**D**). (**E** and **F**) Percentage of time sleeping during the light phase. The KO+vehicle and KO+FMRP groups showed significant reductions in sleep in the male mice (**E**). In the female mice, KO+vehicle group showed significant reduction in the first day, which was reversed in the KO+FMRP group (**F**). ^#^ and ^$^ denote statistically significant differences (*P* < 0.05 for 1 symbol, *P* < 0.01 for 2 symbols, *P* < 0.001 for 3 symbols, and *P* < 0.0001 for 4 symbols) among the 3 treatment groups using 2-way repeated measures ANOVA by treatment and by treatment × time, respectively. * denotes statistically significant differences (*P* < 0.05) between treatment groups using post hoc Tukey’s multiple-comparison test. WT+vehicle, *n* = 14 males and 10 females; KO+vehicle, *n* = 20 males and 11 females; KO+FMRP, *n* = 12 males and 10 females.

To assess the effect of AAV-FMRP treatment on hyperactivity, mice in the 3 treatment groups were tested in the open field test for 20 minutes. Significantly increased distances traveled were observed in the KO+vehicle and the KO+FMRP groups compared with WT+vehicle group in both male and female mice (Supplemental Figure 4). No differences between the KO+vehicle and KO+FMRP groups were observed. The open field test is a short-duration photobeam-based test and has been used traditionally to measure hyperactivity in rodents within a novel setting. We also assessed circadian locomotor activity by video recording the mice for 3 days in a home cage setting and tracking the distance travelled using the neural network DeepLabCut (DLC). This method allowed us to evaluate the activity of the mice for a longer duration in a home cage environment. To ascertain whether the results from the 2 methods were comparable, the activity of the first 3 hours after being placed into the video recording apparatus was evaluated (Figure 5, A and B). As expected, significant increases in distances traveled were observed in the first hour, with significant differences between the WT+vehicle group and the KO+vehicle group in the male mice, and between the WT+vehicle groups and the KO+FMRP groups in the male and female mice, comparable to what was found in the open field test. In the subsequent second and third hours, the activity in all 3 treatment groups decreased as the mice acclimatized to the new environment. However, significantly higher activity was still found in the KO+vehicle group when compared with the WT+vehicle group in the second hour in the male mice (Figure 5A), and in the third hour in the female mice (Figure 5B), while no differences were observed between the WT+vehicle and KO+FMRP groups. This outcome suggested that AAV-FMRP treatment reduced the hyperactivity in the KO mice, which might have reflected elevated anxiety experienced in a novel environment.

To evaluate circadian locomotor activity, mice were video recorded continuously for 3 days, during which locomotor activity (distance traveled) in the light phase only and in the dark phase only (12-hour light/12-hour dark cycle) was measured. In the dark phase, in which mice are naturally more active, no differences in activity were observed among the 3 treatment groups in both male and female mice (data not shown). In the light phase, a significant increase in activity was observed in the male KO+vehicle group compared with the WT+vehicle group on day 1 and day 2 but not with the KO+FMRP group (Figure 5C). In the female mice, the same trend was observed on day 1, though the difference between the KO+vehicle and WT+vehicle groups was not statistically significant (*P* = 0.056; Figure 5D). Since mice are nocturnal animals, further analysis was performed to evaluate whether the increase in light phase activity in *Fmr1*-KO mice was related to reduced sleep. Sleep time was assessed using an algorithm based on inactivity as described by Pack et al. (20), and *Fmr1*-KO mice have been found previously to have reduced sleep using the same method (21). In the male mice, a significant decrease in sleep time was found in both KO+vehicle and KO+FMRP groups in comparison with the WT+vehicle group on all 3 days (Figure 5E). In the female mice, a significant decrease in sleep was found in the KO+vehicle compared with the WT+vehicle, and this difference was not observed in the KO+FMRP group (Figure 5F). These findings suggest that the light phase hyperactivity found in the *Fmr1*-KO mice may be related to reduced sleep and that this deficit was improved by AAV-FMRP gene therapy treatment.

### Correlation between FMRP expression and efficacy.

**Figure 6:**
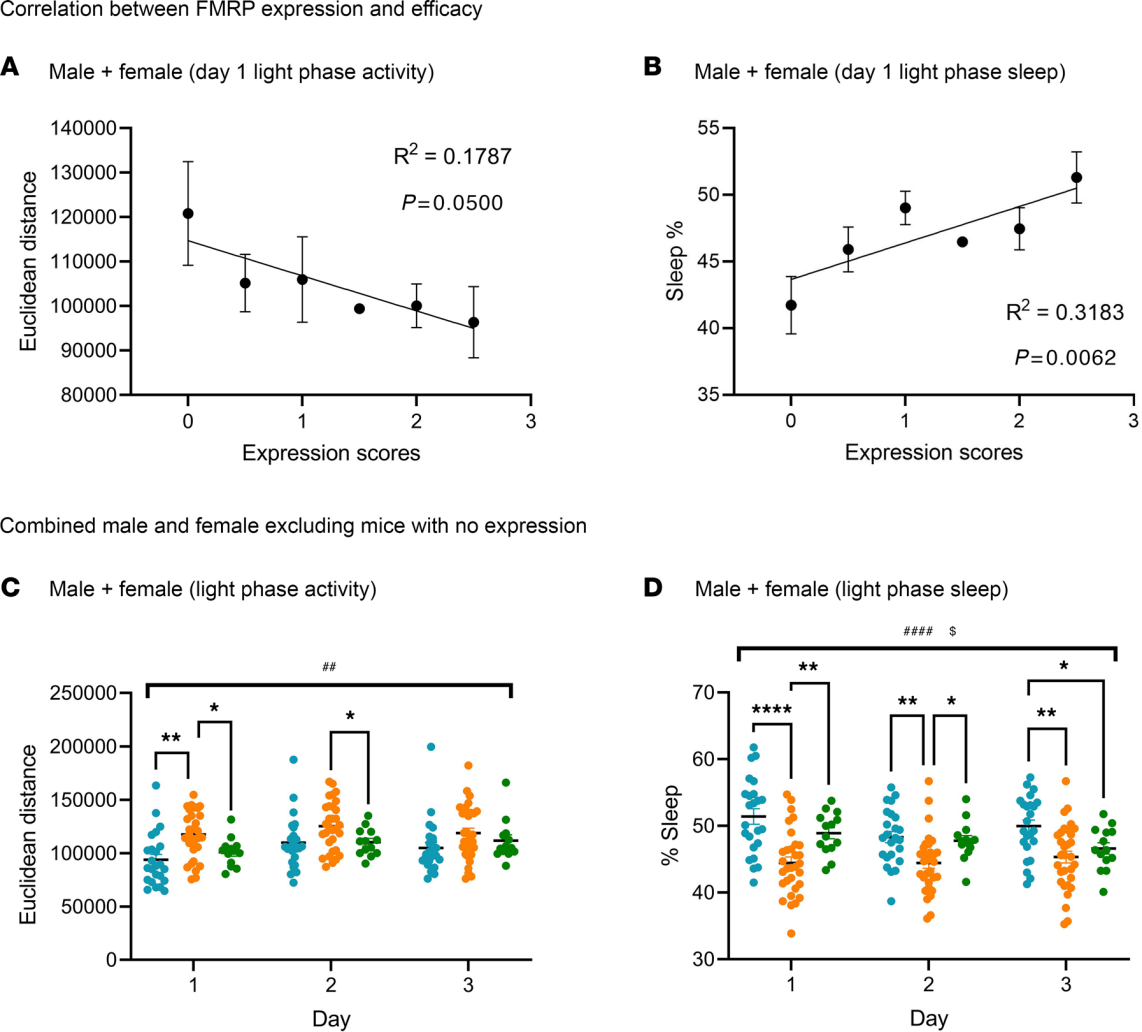
Correlation between FMRP expression level and therapeutic efficacy in light phase activity and sleep. (**A** and **B**) FMRP expression scores (mean ± SEM) correlate with circadian locomotor activity in the light phase (**A**) and sleep time (**B**) in the KO+FMRP group (male and female combined). *R*^2^ refers to goodness of fit by simple linear regression, and *P* value refers to whether the slope is significantly non-zero using the *F* test. Number of mice with FMRP expression score equals to 0 (*n* = 4), 0.5 (*n* = 5), 1 (*n* = 4), 1.5 (*n* = 1), 2 (*n* = 5), 2.5 (*n* = 3). (**C** and **D**) KO+FMRP group were significantly different from the KO+vehicle group in light phase activity (**C**) and sleep time (**D**) during the first and second day of recording after mice with no FMRP expression in the KO+FMRP group were excluded from analysis (male and female combined). ^#^ and ^$^ denote statistically significant differences (*P* < 0.05 for 1 symbol, *P* < 0.01 for 2 symbols, and *P* < 0.0001 for 4 symbols) among the 3 treatment groups using 2-way repeated measures ANOVA by treatment and by treatment × time, respectively. * denotes statistically significant differences (*P* < 0.05) between treatment groups using post hoc Tukey’s multiple-comparison test. WT+vehicle, blue dots, *n* = 24; KO+vehicle, orange dots, *n* = 31; KO+FMRP, green dots, *n* = 13.

At the end of the behavioral analyses approximately 3 months after injection, all mice in the KO+FMRP treatment group (120 mice) were collected and analyzed for brain FMRP transgene expression via tissue sectioning and immunostaining. Each mouse was scored based on the level of expression (see Supplemental Figure 5 for a description of the scoring matrix). To investigate the relationship between FMRP expression and therapeutic efficacy in the context of motor hyperactivity and impaired sleep, simple linear regression was performed between the FMRP expression scores and light phase activity, and FMRP expression scores and sleep time (Figure 6, A and B), in KO+FMRP mice (male and female combined). This analysis was performed on the results from day 1, when the hyperactivity and sleep deficit were most prominent (Figure 5). Light phase activity was negatively correlated to FMRP expression (Figure 6A) while sleep time was positively correlated to FMRP expression (Figure 6B). Both relationships were significantly non-zero by the *F* test (*P* < 0.05). These results showed that the efficacy of the AAV-FMRP treatment in reducing hyperactivity and sleep deficit was proportionally related to the level of FMRP expression in the *Fmr1*-KO mice.

After demonstrating that FMRP expression was correlated to efficacy, male and female mice in the KO+FMRP group with little or no FMRP expression (score < 1) were excluded, and the results in light phase activity and sleep time were reanalyzed (male and female combined; Figure 6, C and D). Light phase activity of the KO+vehicle group was significantly higher than WT+vehicle and KO+FMRP groups on day 1 and was also higher than the KO+FMRP group on day 2 (Figure 6C). For sleep time, KO+vehicle group was significantly lower than both WT+vehicle and KO+FMRP groups on day 1 and 2 (Figure 6D). Excluding mice with little or no FMRP expression further highlighted the efficacy of AAV-FMRP gene therapy on correcting hyperactivity and sleep deficits.

### EEG.

**Figure 7:**
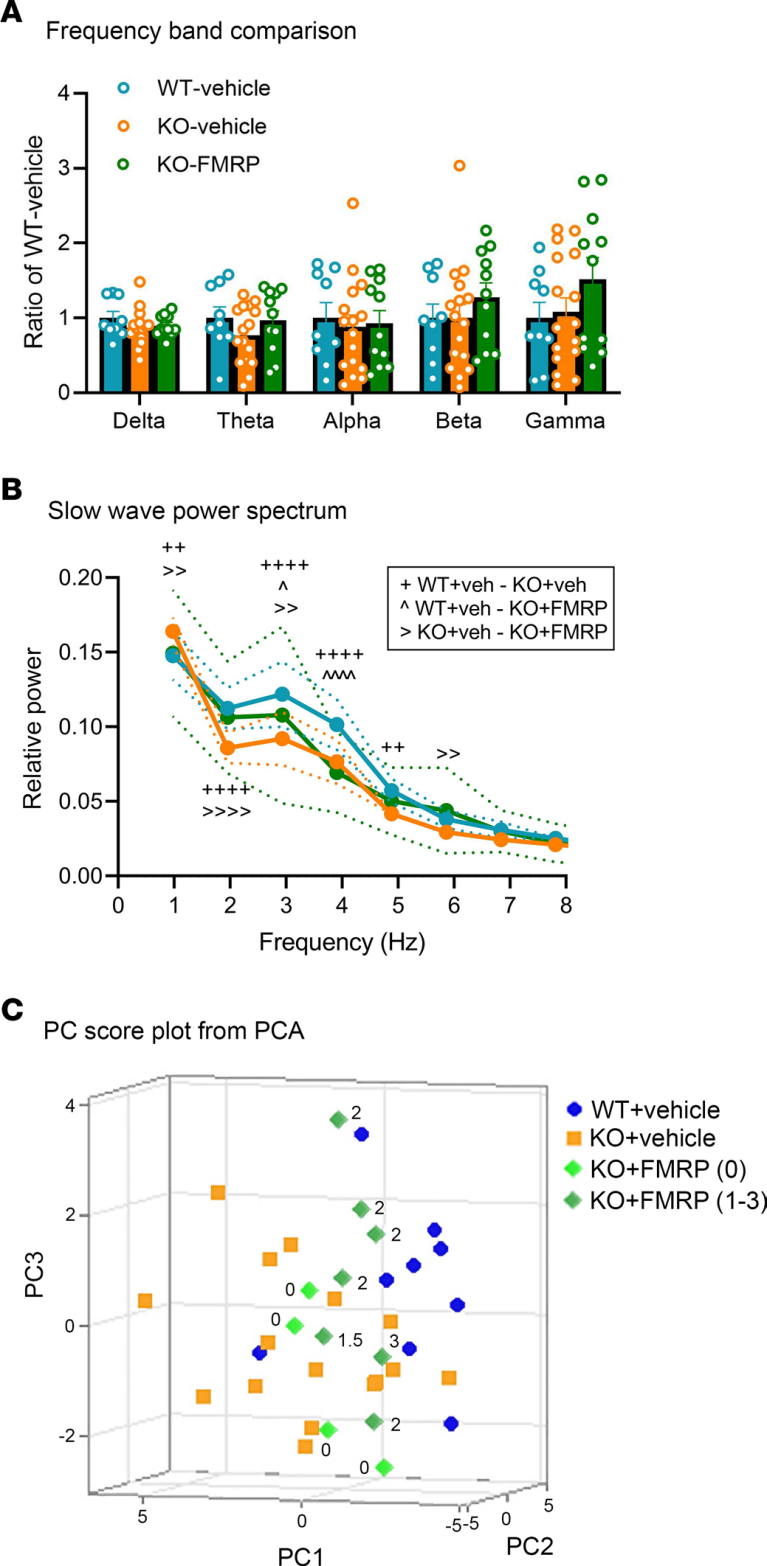
Results of the EEG recordings and principal component analysis. (**A**) EEG frequency band comparison between the 3 treatment groups. (**B**) Comparison of slow-wave power spectrum revealed a significant decrease in the 2–3 Hz delta frequency range in the KO+vehicle group compared with the WT+vehicle group. This deficit was rescued in the KO+FMRP group. ^+^, ^^^, ^>^ represent statistically significant difference (*P* < 0.05 for 1 symbol, *P* < 0.01 for 2 symbols, and *P* < 0.0001 for 4 symbols) between the WT+vehicle and KO+vehicle groups, the WT+vehicle and KO+FMRP groups, and the KO+vehicle and KO+FMRP groups, respectively. (**C**) 3D scatterplot of PC1, PC2, and PC3 scores of male mice that underwent both circadian locomotor activity and EEG recording. The FMRP expression score for each mouse in the KO+FMRP group is shown next to each data point. WT+vehicle (*n* = 9); KO+vehicle (*n* = 16); KO+FMRP (*n* = 11).

EEG recordings were performed in the male mice following circadian locomotor activity recording. Abnormalities in EEG patterns have been consistently reported in patients with FXS and *Fmr1*-KO mice and rats (4, 22–24). In our previous study in *Fmr1*-KO rats, this increase was only observed in males and not in females; therefore, EEG recordings were performed only in the male mice in this study (24). Frequency band comparisons of EEG spectral power during immobility among the 3 treatment groups are shown in Figure 7A. Higher gamma frequency band power was observed in the KO+FMRP group compared with the WT+vehicle and KO+vehicle groups, but the difference was not statistically significant. Comparison of the full power spectrum found a significant difference among the 3 groups by treatment (2-way ANOVA; Supplemental Figure 6). Post hoc Tukey’s multiple-comparison test revealed a significant decrease in slow-wave activity (2–5 Hz) in the KO+vehicle group compared with the WT+vehicle group. This decrease was reversed in the KO+FMRP group in the 2–3 Hz frequency range (Figure 7B). This result was consistent with our previous study in *Fmr1*-KO rats (4), where a decrease in delta wave activity (1–3 Hz) was observed in *Fmr1*-KO rats during sleep, and this decrease was rescued by AAV-FMRP gene therapy.

### Multivariate analysis of track 1 male mice.

Principal component analysis (PCA) was performed on the multidimensional data set from the male mice, which underwent both circadian locomotor activity recording and EEG analysis. Three principal components (PCs) were selected by parallel analysis, and together they explained the majority (>75%) of the total variance. A 3D plot showing the PC1, PC2, and PC3 scores of each mouse from the 3 treatment groups is shown in Figure 7C, with the corresponding descriptive and inferential statistics presented in Table 1. The component loadings are shown in Supplemental Table 1. The FMRP expression score of each mouse in the KO+FMRP group is indicated next to each data point in the figure. The WT+vehicle and KO+vehicle groups were neatly discriminated into 2 clusters, revealing the different phenotypes of the 2 groups. In the KO+FMRP group, the mice with no FMRP expression (expression score = 0) clustered closer to the KO+vehicle group, while the mice with higher FMRP expression levels (expression score ≥ 1) clustered closer to the WT+vehicle group. A statistically significant difference (*P* < 0.05) was found between the mean PC1 scores of the WT+vehicle group and the KO+vehicle group but not between the WT+vehicle group and the KO+FMRP group (*P* > 0.05) by post hoc Tukey’s test (Table 1). Thus, multivariate analysis using PCA revealed that AAV-FMRP gene therapy rescued the abnormal phenotype of *Fmr1*-KO mice and that this was associated with the expression of the FMRP transgene.

## Discussion

Because FXS is a neurodevelopmental disorder, very early postnatal treatment with AAV-FMRP is expected to provide superior clinical benefit compared with delayed treatment. Early postnatal treatment at PND 2 and 3 was also used here because previous studies have shown a more widespread diffusion of AAV-FMRP after intra-CSF administration in the brain at this early age than older mice and rats (3, 4, 25). Nevertheless, future studies in mice injected at later time points may be informative. Although the PND 2 or 3 mouse is roughly equivalent to a third trimester human pregnancy, some studies (26, 27), but not all (28), have demonstrated good diffusion after i.t. injections of AAVs into adult or juvenile nonhuman primates (NHPs). Intra–cisterna magna injection into the CSF is an alternative route that may convey better brain transduction compared with i.t. injection via lumbar puncture in primates (28–30).

Clinical translatability is crucial in drug development and was an important facet of the design of the vector, route of administration, and safety testing of this study. In a previous study, strong overexpression of FMRP in neurons (>2.5× WT levels) led to deleterious effects (2). The JeT promotor used here is a relatively weak ubiquitous promotor and was selected over stronger neuronal specific promotors to minimize potential overexpression. A JeT promotor–driven vector is currently being tested in an AAV gene therapy clinical trial for giant axonal neuropathy (ClinicalTrials.gov Identifier: NCT02362438) and CLN7 Batten disease (NCT04737460).

While the vector was administered as a fixed dose per animal, the extrapolated dose per body weight used in this study was 1.13 × 10^14^ vg/kg. This is within the range of the AAV doses administered in previous clinical trials with infants or young children. For example, in the clinical trials for Zolgensma, the FDA-approved gene replacement therapy for spinal muscular atrophy, infants (1–8 months old) were treated at doses of 6 × 10^13^ vg/kg or 2 × 10^14^ vg/kg (31). The AAV-FMRP dose used in this study was the highest given among previous gene therapy studies in *Fmr1*-KO mice and may have contributed to the improved efficacy observed.

Recently, serious side effects have been associated with high doses of AAVs. Tragically, 4 boys (<5 years old) have died in a clinical trial for X-linked myotubular myopathy, with 3 having received the high dose of 3.5 × 10^14^ vg/kg and 1 the lower dose of 1.3 × 10^14^ vg/kg (32). Severe toxicities have also been observed in 14-month-old juvenile NHPs and piglets (7–30 days old) with AAV expressing human survival motor neuron protein at a dose of 2 × 10^14^ vg/kg (33). It is important to note that in those studies, AAVs were administered by systemic intravenous injections, and those types of severe adverse effects have not been associated with intra-CSF administration of AAV. Since immune responses to the AAV vector have been thought to be the cause of some of the more serious adverse events, toxicity could have been lower when the AAV vectors were administered to immune-privileged sites such as the CNS (34, 35). In the current study, no significant adverse effects were observed in serum markers, liver pathology, and in behavioral assays, even when the transgene was overexpressed in WT mice (Supplemental Figure 2). Also, FMRP is naturally expressed throughout the body, including the liver, and so expression in peripheral tissues could be beneficial in the case of FXS. Administration of AAV vectors has also been shown to induce dorsal root ganglion (DRG) pathology in NHPs and piglets (33). In an aggregated analysis from 33 studies including more than 200 NHPs, the majority of the DRG pathology observed following intra-CSF injections was minimal to moderate and clinically asymptomatic (36). In one study where infant NHPs were administered AAV9 vectors, no DRG histopathological abnormalities were observed 4 years after injections (37). In our study, although we did not directly examine DRG, no abnormalities in motor coordination were observed in the rotarod and open field tests. In future clinical studies, monitoring sensory neuropathies may be needed to eliminate concerns of potential DRG neuropathies.

The *FMR1* gene undergoes extensive alternative splicing. Here, human isoform 17 was chosen for efficacy testing because it is a highly abundant FMRP isoform in the human brain (9, 38). The design of the AAV-FMRP vector imposed the practical size limitations of a self-complementary genome (~2,200 nt of foreign DNA), leaving only approximately 300 nt of space to include regulatory elements outside the coding region of FMRP (e.g., promoter and polyA). The original G-quartet motif sequence in the FMRP mRNA was deliberately left unaltered to allow interaction between the mRNA and the FMRP RGG domain for posttranscriptional regulation. Conceptually, regulatory controls are missing compared with the endogenous locus. Empirically, however, enough of these controls appear to remain intact to approximate the native expression pattern of FMRP and impart significant therapeutic benefits. Among CNS delivery routes, the i.t route is the least invasive in comparison with intraventricular or intra–cisterna magna injections. Transgene expression after i.t. delivery in this study closely mimicked the CNS expression pattern of other AAVs after intra-CSF administration in NHPs with robust expression in the primate cortex, brainstem, and cerebellum and lower levels in subcortical structures such as striatum and thalamus (39). This may be important because in our previous studies, intraventricular or intra–cisterna magna injections showed a different expression pattern in mice and rats with robust expression in the cortex and some subcortical forebrain structures and less in the brainstem and cerebellum (2, 4). This suggests that i.t. injection may be the most translatable CNS delivery route from rodents to humans. Expression in the brainstem may have been crucial for the therapeutic effects observed in this study since this structure is known to play important roles in the regulation of sleep and the generation of AGSs (18, 40, 41).

In the mouse model of FXS and in human patients, neuronal and sensory hypersensitivity is a commonly reported endophenotype (42, 43). In both humans and mice, this may be reflected as an enhanced startle response and increased EEG resting gamma power (22, 23). In *Fmr1*-KO mice, AGSs are also a manifestation of neuronal/sensory hypersensitivity, with up to 70%–80% of *Fmr1*-KO mice reported as experiencing AGS (Figure 3A) (19). Despite such a strong endophenotype, treatment with AAV-FMRP was able to rescue this behavior during the peak sensitivity period (PND 27–31). We believe this is the first time AGS has been reported to be rescued in an FXS model with gene therapy. The robust suppression of AGSs may have been due to the use of a CNS-dominant isoform of FMRP, the overall vector design, and/or injection via the i.t. route, which resulted in improved FMRP expression in caudal areas of the brain, such as the inferior colliculus and brainstem, which are involved in the generation of AGSs (18, 44). While patients with FXS do not experience AGSs, up to 20% experience generalized epileptic seizures. The rescue of AGS demonstrated here is representative of the potential of AAV-FMRP treatment to correct abnormal neuronal circuitry and life-altering hypersensitivity in people with FXS (40, 45, 46).

Approximately one-third of children with FXS have been reported to have sleep difficulties that encompass problems falling asleep and frequent nighttime awakenings (47). In this study, gene therapy reversed the sleep deficit in *Fmr1*-KO mice in activity/inactivity-based sleep analysis and normalized slow-wave activity in EEG recordings (Figure 5, E and F, and Figure 7B). Slow delta wave activity is indicative of slow-wave sleep. We previously reported similar results in the *Fmr1*-KO rat where a reduction in slow-wave power during sleep was also rescued by AAV-FMRP treatment (4, 24). These results could be translatable as sleep disturbances have been reported in children with FXS in polysomnographic studies with EEG recordings, as well as actigraphy studies where awake and sleep parameters were determined by an algorithm based on movement data collected from a wrist-worn monitor (12–14).

Inconsistent findings in conventional behavioral tests have been widely reported in *Fmr1*-KO mice (48). It is important to note that the overall phenotype of the *Fmr1*-KO mouse consists of multiple subtle endophenotypes, which can complicate behavioral assessments. For example, the hyperactive phenotype of *Fmr1*-KO mice has been reported to be a confounding factor in assessing sustained attention (48). In the current study, multivariate PCA was performed using the data from mice that underwent multiple tests (circadian locomotor recording and EEG analysis). Data sets without statistically significant differences were also included, such as dark phase activity and EEG relative power. The results showed a clean discrimination between WT+vehicle groups and the KO+vehicle group, representing the distinct overall behavioral phenotypes of the 2 genotypes (Figure 7C). Remarkably, AAV-FMRP–treated mice with FMRP expression clustered with the WT+vehicle group, while mice with little or no expression clustered with the KO+vehicle group, demonstrating the rescue of the overall abnormal phenotype by the AAV-generated transgene. Our results suggest that multivariate analysis such as PCA can facilitate behavioral analyses of animal models with subtle endophenotypes.

We were able to establish, through linear correlations and PCA, a direct proportional relationship between transgene expression and efficacy, demonstrating that behavioral abnormalities in *Fmr1*-KO mice could be corrected by increasing the number of cells expressing the FMRP protein. Removal of mice with no or low FMRP expression from the efficacy analysis further delineated the difference between the KO+vehicle and KO-FMRP groups in hyperactivity, sleep, and AGSs. Our results are consistent with a previous study where FMRP levels were shown to correlate with the IQs of individuals with FXS (49). Also relevant is the finding that only about 35% of WT levels of FMRP were sufficient to detect behavioral improvement in *Fmr1*-KO mice treated with AAV-FMRP (2), and intriguingly, a similar level of residual FMRP expression in people with FXS was sufficient to attain a mean IQ of 85, the lower bound for normal IQ range (50). Together, these considerations, combined with ongoing efforts to conduct large-scale and widespread prenatal and newborn genetic screening in the population (51–53), will increase the pool of infants identified with the *Fmr1* mutation who would be eligible for early gene therapy treatment.

## Methods

### Overview of study design.

A battery of behavioral tests was performed to evaluate the effects of AAV-FMRP gene therapy in *Fmr1*-KO mice. Different groups of mice were used to minimize the effects of repeated handling and testing. The results were compared among 3 treatment groups: WT+vehicle, KO+vehicle, and KO+FMRP. One group of mice was used for the evaluation of AGSs. A separate group of mice was used for the circadian locomotor activity recording and for EEG recordings. A third group of mice was used for the open field test followed by the fear conditioning test. The identities of the treatment groups were blinded until all the behavioral tests and data analyses were completed.

### Animals.

WT (strain 00664) and *Fmr1*-KO mice (strain 003025) (C57BL/6 background) were purchased from The Jackson Laboratory. Mice were housed in groups of 3–4 on a 12-hour light/12-hour dark cycle with food and water ad libitum. All behavioral experiments were performed between 12 and 6 pm.

### AAV vector.

The AAV-FMRP vector was produced by the University of Texas Southwestern Translational Gene Therapy Core (TGTC) and formulated in phosphate-buffered saline containing 5% d-sorbitol and 0.001% pluronic F-68. It was produced by triple transfection of HEK293T cells, followed by cell lysis and recovery of recombinant AAV virus from the cells and media. Purification was through filtration, affinity chromatography, and anion exchange chromatography using methods developed at the TGTC. The vector was titered by quantitative PCR directed to the *FMR1* transgene, using a highly purified linearized plasmid standard. The release testing results are provided as Supplemental Figure 7.

### Vector administration.

For the behavioral efficacy experiments, AAV-FMRP vector (3.23 × 10^13^ vg/mL) or vehicle (1× phosphate-buffered saline containing 5% d-sorbitol and 0.001% pluronic F-68) was administered to mouse pups at PND 2 or 3 via lumbar i.t. injection. Injections were performed using a 30-gauge needle (7803-07, Hamilton Company) with a 50 μL syringe (7637-01, Hamilton Company). During the injection, the mouse pup was held gently by the pelvic girdle in a prone position while the needle was inserted into the L5–L6 intervertebral space at a 30° angle. A total volume of 7 μL was injected over 15 seconds, after which the needle was left in place for an additional 15 seconds. The dose for the KO+FMRP treatment group was 2.3 × 10^11^ vg per mouse. For the RNAscope and safety experiments in WT mice, mouse pups were injected with 1.3 × 10^11^ vg or 5.0 × 10^11^ vg of the vector by lumbar i.t. injections at PND 7–10.

### RNAscope.

The ACD RNAscope 2.5 HD Assay Kit 322360 was used. Five μm section slides were deparaffinized by xylene followed by 100% ethanol, then incubated with hydrogen peroxide for 10 minutes at room temperature and washed with distilled water. Antigen retrieval was performed by boiling slides in 1× Target Retrieval solution (ACD 322000) for 10 minutes, washing with distilled water, and then dehydrating with ethanol and air-drying. Protease Plus was added to each section, incubated at 40°C for 30 minutes, and washed with distilled water. The slides were incubated with a custom-made h*FMR1*-Codon-C1RNAscope probe in a HybEZ oven for 2 hours at 40°C and washed with 1× wash buffer, followed by incubating with AMP 1–6 for 30 or 15 minutes and the use of the RNAscope 2.5 HD Detection Kit protocol. The G-quartet motif in the RGG domain of the endogenous *FMR1* sequence was not codon-optimized to ensure retention of FMRP binding to *FMR1* mRNA. The slides were then incubated with kit-provided RED solution for 10 minutes, counterstained with Mayer’s hematoxylin, and imaged with an Aperio ImageScope; histology images were analyzed using custom analysis settings in the HALO image analysis platform (HALO2.2, Indica Labs).

### Western blot.

Western blots were carried out as previously described, using 10% SDS-PAGE gels and transfers to nitrocellulose (4). The blots were probed with anti-FMRP antibody (MMS-5232, BioLegend, 1:1,000) and appropriate horseradish peroxidase secondary antibody (goat anti-mouse IgG-HRP, 111-035-003, Jackson ImmunoResearch, 1:2,000). The blots were visualized on a Bio-Rad ChemiDoc imaging system.

### Immunohistochemical analyses.

For immunohistochemistry, frozen mouse brains were collected and sectioned in the sagittal plane at 30 μm on a cryostat (Leica), then immunostained as previously described (4). The following primary antibodies were used: mouse anti-FMRP (MMS-5232, BioLegend, 1:500), rabbit anti-FMRP (ab17722, Abcam, 1:2,000), mouse anti-NeuN (MAB377, MilliporeSigma, 1:1,000), rabbit anti-GAD65/67 (ab183999, Abcam, 1:500), rabbit anti-Sox9 (ab185230, Abcam, 1:2,000), and rabbit anti-S100B (S2532, Abcam, 1:2,000), diluted in blocking solution. The following secondary antibodies were used (all purchased from Thermo Fisher Scientific, 1:2,000 to 1:3,000): Alexa Fluor 594–conjugated anti–rabbit IgG (A-11037), Alexa Fluor 488–conjugated anti–rabbit IgG (A32731), Alexa Fluor 594–conjugated anti–mouse IgG (A-11032), and Alexa Fluor 488–conjugated anti–mouse IgG (A-11029), diluted in blocking solution for 2 hours. Samples were mounted on glass slides using ProLong Gold Antifade Mountant (P36930, Life Technologies).

The brains of all KO+FMRP behavior and electrophysiology test animals were collected at the end of each experiment, and brain sections were prepared and immunostained for FMRP. Images were captured with an LSM710 confocal microscope (ZEISS) using a 20× objective lens, or using a BioTek Cytation 5 Cell Imaging Multi-Mode Reader (Bio-Rad) using a 4× objective lens. Microscope settings (pinhole, gain, and contrast) were kept constant for all images in each experiment. Images were binned according to a CNS FMRP expression rubric (Supplemental Figure 3) as scored by 2 independent researchers whose scores were then averaged.

For cell type coverage and specificity quantification experiments, confocal images of a fixed area of the motor cortex were taken with an LSM710 confocal microscope using a 20× objective lens. The number of cells positive for FMRP, each cell type marker, and for both FMRP and each cell type marker (double-labeled) were counted. Coverage was defined as (# double-labeled cells)/(# cells of a given cell type) × 100%. Specificity was defined as (# double-labeled cells)/(# FMRP-positive cells) × 100%. Microscope settings (pinhole, gain, and contrast) were kept constant for all the images in each experiment. Image analysis and quantification were carried out using FIJI (54).

### AGS testing.

For AGS testing, the apparatus consisted of a plastic mouse cage (28 × 17 × 14 cm) with a 125 dB sound source (Piezo siren, electrosonic; Piezo Technologies) attached to the lid and extending 5 cm down into the cage, placed inside a larger soundproof container, and monitored by video recording. Mice (27–31 days old) were placed individually into the testing apparatus and were allowed to explore for 2 minutes, after which the sound source was activated for 3 minutes. Seizure activity was observed and scored using a seizure severity score as follows: wild running – 1; clonic seizure – 2; tonic seizure – 3; status epilepticus/respiratory arrest/death – 4. Animals were considered to have had a seizure if the seizure severity score was greater than 1. Only animals with an FMRP expression score of more than 1 were used in the final analyses (Supplemental Figure 3).

### Fear conditioning test.

Fear conditioning was performed with mice at PND 71–78 as previously described (24). Briefly, animals were tested in a soundproof chamber using a mouse fear conditioning system with mouse cage (46000, Ugo Basile). Data and video were recorded using the accompanying ANYmaze software (v7.1, Stoelting Co.). All testing was performed between 12 and 6 pm with white noise and lighting in the chamber. Fear conditioning consisted of a 2-day protocol as described (Figure 4A), with conditioning/training on the first day, and the second day consisting of measuring of the conditioning response to the original conditioned context (context A), to a novel context (context B), and to the conditioned tone presented in context B, for 3 minutes each. Only animals with an FMRP expression score of more than 1 were used in the final analyses (Supplemental Figure 3).

### Open field test.

The open field test was conducted using the VersaMax Animal Activity Monitor (Omnitech Electronics). Mice (PND 50–55) were acclimated to the test room for 45–60 minutes before being placed in a 40 × 40 cm arena under low light conditions (2–3 lux). The testing apparatus was enclosed inside an environmental control chamber (Omnitech Electronics) to minimize noise distractions. Mice were allowed to freely explore for 20 minutes, and total distance traveled was tabulated using the VersaMax Analyzer software.

### Circadian locomotor activity and sleep.

Mice (PND 59–65) were video recorded for 3 consecutive days to evaluate circadian locomotor activity. The procedures were adapted from the methods as described by Wong et al. (24). The recording apparatus consisted of two 28 × 17 × 13 cm transparent plastic mouse cage bottoms enclosed by 55 cm tall panels made of transparent acrylic. The 2 cages were separated by a transparent acrylic divider. Two cage mates were placed separately into the 2 compartments with food and water ad libitum. To minimize stress from being single-housed during the recording, the 2 compartments were not sealed to allow the scents of the mice to flow between the compartments. Videos were recorded at 2 frames/s by a Raspberry Pi 3 model B microcomputer (RS Components, Ltd.) equipped with a Smraza 5MP 1080p OV5647 Video Webcam Night Vision camera located on top of the cages. DLC (version 2.0) was used to analyze the videos to obtain the *X*, *Y* coordinates of the mice, and the distance traveled was calculated from the coordinates as Euclidean distance in pixels by applying the Pythagorean theorem (24). Sleep was determined based on activity/inactivity as described by Pack et al. (20). A mouse was considered to be inactive when its velocity was less than 3 pixels/s. Any episodes of continuous inactivity at least 40 seconds were defined as sleep. Over 90% agreement was found when a simultaneous comparison between this method and sleep assessment by EEG/electromyography was performed in the same mice (20).

### EEG.

The methods for the construction and implantation of the electrodes, EEG recording, and data analysis were performed as described by Wong et al. (24). Briefly, 2 bipolar electrodes were placed in the frontal lobes at the following stereotaxic coordinates relative to the bregma (+2.7 anterior/posterior, ±1.5 medial/lateral) (at a depth of ~1.5 mm) according to *The Mouse Brain in Stereotaxic Coordinates* by Paxinos and Franklin (55). A reference electrode was placed in the occipital lobe epidurally at (–4.0, –1.5). These coordinates were adapted from Lovelace et al. (22). Recordings were performed 5–10 days after implantation surgery at PND 77–91 with a differential AC amplifier (Model 1700, A-M Systems) and digitized by Digidata 1550B data acquisition system (Axon Instruments, Molecular Devices). Data acquisition and analysis were performed using pCLAMP software version 11 (Axon Instruments, Molecular Devices). The mice were recorded in an acrylic chamber for 3 hours accompanied by video recording. Artifact-free epochs (>30 seconds) during immobility were selected based on video recording, and Fast Fourier Transform was run to calculate spectral power from 0 to 100 Hz. Relative power for each frequency band (delta: 1–4 Hz; theta: 4–8 Hz; alpha: 8–12 Hz; beta: 12–30 Hz; and gamma: 30–100 Hz) was calculated by dividing the spectral power of a given band by the total spectral power (0–100 Hz). A 60 Hz notch filter was used to eliminate line noise, and 50 to 70 Hz were excluded from all analyses.

### PCA.

PCA was performed on the data set collected from the male mice that underwent circadian locomotor activity monitoring and EEG recording using GraphPad Prism 9. Fourteen variables from circadian locomotor activity and EEG recording were input for analysis including distance traveled during light phase (day 1, 2, and 3) and dark phase (day 1, 2, and 3), total percentage of sleep time during light phase (day 1, 2, and 3), and relative EEG spectrum powers (delta, theta, alpha, beta, and gamma). Data were standardized to a mean of 0 and a standard deviation of 1. PCs were selected by parallel analysis, and the resulting descriptive and inferential statistics are shown in Table 1. The component loadings are shown in Supplemental Table 1. The PC scores from PC1, PC2, and PC3 were compared using 1-way ANOVA and post hoc Tukey’s multiple-comparison test. The method of incorporating PCA and the comparison of PC scores into the analysis of behavioral data was adapted from Giuliani (56). The 3D scatterplot was drawn using Minitab statistical software.

### Safety testing — histology.

WT mice injected at PND 7–10 were sacrificed at 4 weeks or 12 months postinjection. Tissues were collected and drop-fixed in 10% neutral buffered formalin for 24 hours and then placed in 70% ethanol for storage. The tissues were trimmed into cassettes and embedded into paraffin. One hematoxylin and eosin–stained slide was produced from each cassette. All slides were evaluated under a blinded protocol by a veterinary pathologist at the University of Texas Southwestern. Samples of liver from WT and KO mice were also analyzed by the Toronto Centre for Phenogenomics (Toronto, Ontario, Canada). Liver samples were stained with hematoxylin and eosin and analyzed by a trained histopathologist for signs of inflammation.

### Safety testing — serum toxicity assay.

Blood was collected from the heart at necropsy. Blood was allowed to clot at room temperature for 2 hours. The clotted material was removed by centrifugation at 1,200*g* for 10 minutes at 4°C. The supernatant (serum) was transferred to a new tube and frozen immediately at −80°C until the test. Serum analyses were conducted by the University of Texas Southwestern Metabolic Phenotyping Core and the Toronto Centre for Phenogenomics.

### Statistics.

For the AGS experiments, Fisher’s exact test was used to compare seizure incidences between the 3 treatment groups, while seizure time was analyzed using 1-way ANOVA followed by post hoc Tukey’s multiple-comparison test, and seizure level was analyzed using the Kruskal-Wallis test followed by post hoc Dunn’s multiple-comparison test. Fear conditioning used a 1-way ANOVA followed by Tukey’s post hoc. For the other behavioral experiments, the 3 treatment groups were compared using 2-way repeated measures ANOVA with time and treatment as factors, followed by post hoc Tukey’s multiple-comparison test. Simple linear regression was performed to analyze the relationship between transgene expression and efficacy in circadian locomotor activity or sleep. Two-tailed Student’s *t* test was used to compare transgene expression coverage and specificity between the WT+vehicle and KO+FMRP groups in immunohistochemical analyses. *P* < 0.05 was considered statistically significant. Error bars represent SEM in all figures. All statistical analyses were performed using GraphPad Prism 9.

### Study approval.

All animal procedures at the University of Toronto were approved by the University of Toronto animal care committee; all animal procedures in WT mice conducted at the University of Texas Southwestern were approved by the University of Texas Southwestern IACUC.

## Author contributions

The order of the authors was established based on the relative contributions of each author. DRH, SJG, HW, AWMH, SR, CS, and HRK conceived the project; HW, AWMH, HRK, SJL, and JZ performed the experiments; DRH, HW, AWMH, HRK, and SJL analyzed the data; and DRH, HW, and AWMH wrote the manuscript with input from all the other authors. HW and AWMH are co–first authors of this paper, have contributed equally to the study, and have alternated the order of first authorship in publications on the FXS gene therapy project.

## Supplementary Material

Supplemental data

## Figures and Tables

**Table 1:**
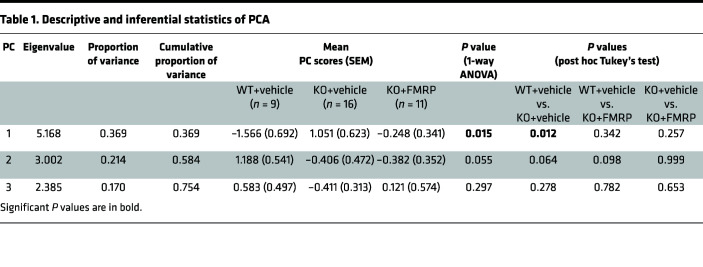
Descriptive and inferential statistics of PCA

## Version 1 06/08/2023

Electronic publication

## References

[1] Zeier Z (2009). Fragile X mental retardation protein replacement restores hippocampal synaptic function in a mouse model of fragile X syndrome. Gene Ther.

[2] Arsenault J (2016). FMRP expression levels in mouse central nervous system neurons determine behavioral phenotype. Hum Gene Ther.

[3] Gholizadeh S (2014). Reduced phenotypic severity following adeno-associated virus-mediated Fmr1 gene delivery in fragile X mice. Neuropsychopharmacology.

[4] Hooper AWM (2021). Gene therapy using an ortholog of human fragile X mental retardation protein partially rescues behavioral abnormalities and EEG activity. Mol Ther Methods Clin Dev.

[5] Jiang Y (2022). Gene therapy using human FMRP isoforms driven by the human *FMR1* promoter rescues fragile X syndrome mouse deficits. Mol Ther Methods Clin Dev.

[6] Brackett DM (2013). FMR1 transcript isoforms: association with polyribosomes; regional and developmental expression in mouse brain. PLoS One.

[7] Fu XG (2020). Splicing of exon 9a in FMR1 transcripts results in a truncated FMRP with altered subcellular distribution. Gene.

[8] Kirkpatrick LL (1999). Alternative splicing in the murine and human FXR1 genes. Genomics.

[9] Pretto DI (2015). Differential increases of specific FMR1 mRNA isoforms in premutation carriers. J Med Genet.

[10] McCarty DM (2001). Self-complementary recombinant adeno-associated virus (scAAV) vectors promote efficient transduction independently of DNA synthesis. Gene Ther.

[11] Deverman BE (2018). Gene therapy for neurological disorders: progress and prospects. Nat Rev Drug Discov.

[12] Carotenuto M (2019). Polysomnographic findings in fragile X syndrome children with EEG abnormalities. Behav Neurol.

[13] Miano S (2008). Sleep phenotypes of intellectual disability: a polysomnographic evaluation in subjects with Down syndrome and fragile-X syndrome. Clin Neurophysiol.

[14] Wirojanan J (2009). The efficacy of melatonin for sleep problems in children with autism, fragile X syndrome, or autism and fragile X syndrome. J Clin Sleep Med.

[15] Schaeffer C (2001). The fragile X mental retardation protein binds specifically to its mRNA via a purine quartet motif. EMBO J.

[16] Goering R (2020). FMRP promotes RNA localization to neuronal projections through interactions between its RGG domain and G-quadruplex RNA sequences. Elife.

[17] Gholizadeh S (2015). Expression of fragile X mental retardation protein in neurons and glia of the developing and adult mouse brain. Brain Res.

[18] Gonzalez D (2019). Audiogenic seizures in the Fmr1 knock-out mouse are induced by Fmr1 deletion in subcortical, VGlut2-expressing excitatory neurons and require deletion in the inferior colliculus. J Neurosci.

[19] Pacey LK (2009). Increased GABA(B) receptor-mediated signaling reduces the susceptibility of fragile X knockout mice to audiogenic seizures. Mol Pharmacol.

[20] Pack AI (2007). Novel method for high-throughput phenotyping of sleep in mice. Physiol Genomics.

[21] Sare RM (2017). Deficient sleep in mouse models of fragile X syndrome. Front Mol Neurosci.

[22] Lovelace JW (2018). Translation-relevant EEG phenotypes in a mouse model of fragile X Syndrome. Neurobiol Dis.

[23] Wang J (2017). A resting EEG study of neocortical hyperexcitability and altered functional connectivity in fragile X syndrome. J Neurodev Disord.

[24] Wong H (2020). Sexually dimorphic patterns in electroencephalography power spectrum and autism-related behaviors in a rat model of fragile X syndrome. Neurobiol Dis.

[25] Gholizadeh S (2013). Transduction of the central nervous system after intracerebroventricular injection of adeno-associated viral vectors in neonatal and juvenile mice. Hum Gene Ther Methods.

[26] Gray SJ (2013). Global CNS gene delivery and evasion of anti-AAV-neutralizing antibodies by intrathecal AAV administration in non-human primates. Gene Ther.

[27] Meyer K (2015). Improving single injection CSF delivery of AAV9-mediated gene therapy for SMA: a dose-response study in mice and nonhuman primates. Mol Ther.

[28] Hinderer C (2020). Adeno-associated virus serotype 1-based gene therapy for FTD caused by GRN mutations. Ann Clin Transl Neurol.

[29] Flotte TR (2022). AAV gene therapy for Tay-Sachs disease. Nat Med.

[30] Taghian T (2020). A safe and reliable technique for CNS delivery of AAV vectors in the cisterna magna. Mol Ther.

[31] Mendell JR (2017). Single-dose gene-replacement therapy for spinal muscular atrophy. N Engl J Med.

[32] Philippidis A (2021). Fourth boy dies in clinical trial of Astellas’ AT132. Hum Gene Ther.

[33] Hinderer C (2018). Severe toxicity in nonhuman primates and piglets following high-dose intravenous administration of an adeno-associated virus vector expressing human SMN. Hum Gene Ther.

[34] Ertl HCJ (2022). Immunogenicity and toxicity of AAV gene therapy. Front Immunol.

[35] Kishimoto TK, Samulski RJ (2022). Addressing high dose AAV toxicity - ‘one and done’ or ‘slower and lower’?. Expert Opin Biol Ther.

[36] Hordeaux J (2020). Adeno-associated virus-induced dorsal root ganglion pathology. Hum Gene Ther.

[37] Hordeaux J (2019). Safe and sustained expression of human iduronidase after intrathecal administration of adeno-associated virus serotype 9 in infant rhesus monkeys. Hum Gene Ther.

[38] Fu X (2015). Alternatively spliced products lacking exon 12 dominate the expression of fragile X mental retardation 1 gene in human tissues. Mol Med Rep.

[39] Kondratov O (2021). A comprehensive study of a 29-capsid AAV library in a non-human primate central nervous system. Mol Ther.

[40] Musumeci SA (2000). Audiogenic seizures susceptibility in transgenic mice with fragile X syndrome. Epilepsia.

[41] Schneider L (2020). Neurobiology and neuroprotective benefits of sleep. Continuum (Minneap Minn).

[42] Miller LJ (1999). Electrodermal responses to sensory stimuli in individuals with fragile X syndrome: a preliminary report. Am J Med Genet.

[43] Rojas DC (2001). Auditory evoked magnetic fields in adults with fragile X syndrome. Neuroreport.

[44] Ribak CE (2017). An abnormal GABAergic system in the inferior colliculus provides a basis for audiogenic seizures in genetically epilepsy-prone rats. Epilepsy Behav.

[45] Bailey DB (2008). , et al. Co-occurring conditions associated with FMR1 gene variations: findings from a national parent survey. Am J Med Genet A.

[46] Berry-Kravis E (2010). Seizures in fragile X syndrome: characteristics and comorbid diagnoses. Am J Intellect Dev Disabil.

[47] Kronk R (2010). Prevalence, nature, and correlates of sleep problems among children with fragile X syndrome based on a large scale parent survey. Sleep.

[48] Kazdoba TM (2014). Modeling fragile X syndrome in the Fmr1 knockout mouse. Intractable Rare Dis Res.

[49] Tassone F (1999). FMRP expression as a potential prognostic indicator in fragile X syndrome. Am J Med Genet.

[50] Kim K (2019). Association between IQ and FMR1 protein (FMRP) across the spectrum of CGG repeat expansions. PLoS One.

[51] Abrams L (2012). Newborn, carrier, and early childhood screening recommendations for fragile X. Pediatrics.

[52] Gutierrez JF (2013). Prenatal screening for fragile X: carriers, controversies, and counseling. Rev Obstet Gynecol.

[53] Kaiser J (2022). Sequencing projects will screen 200,000 newborns for disease. Science.

[54] Schindelin J (2012). Fiji: an open-source platform for biological-image analysis. Nat Methods.

[56] Giuliani A (2017). The application of principal component analysis to drug discovery and biomedical data. Drug Discov Today.

