# Supplementary material for: CNS-dominant human FMRP isoform rescues seizures, fear, and sleep abnormalities in Fmr1-KO mice
Source: JCI Insight. 2023 Jun 8;8(11):e169650. doi: 10.1172/jci.insight.169650 (PMC10393223; doi:10.1172/jci.insight.169650)
Supplement: Supplemental data [file jciinsight-8-169650-s042.pdf]

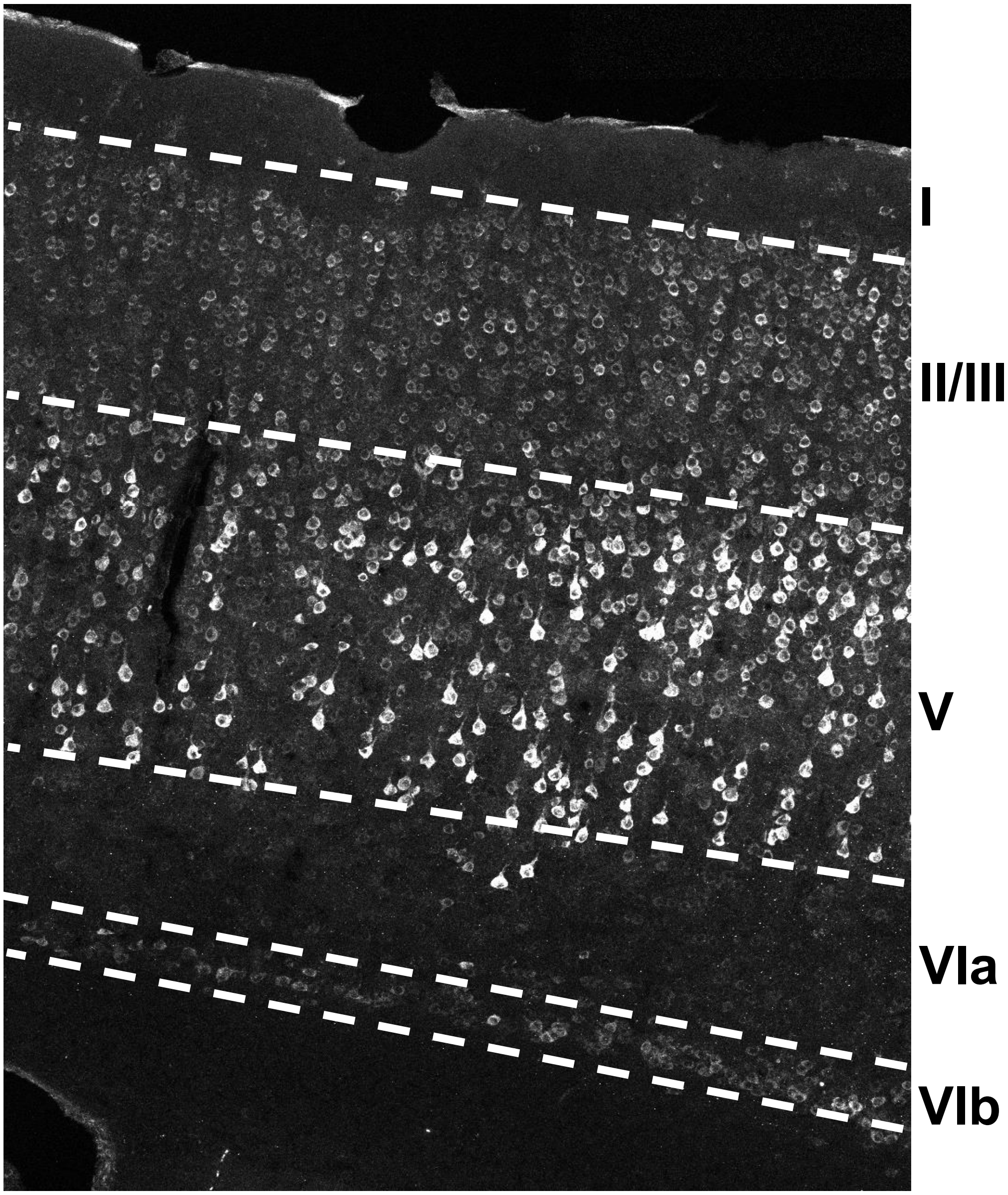

Supplemental Figure 1. Transduction of specific cell layers in the mouse cerebral cortex by AAV-JeT-hFMRPiso17. One month after i.t. injection into a Fmr1 KO mouse at PND-2-3, expression of FMRP was highest in layer V of the motor cortex, followed by moderate expression in layers II/III and VIb. Lower expression was apparent in layers I and VI. Layer IV was not visible in this section.

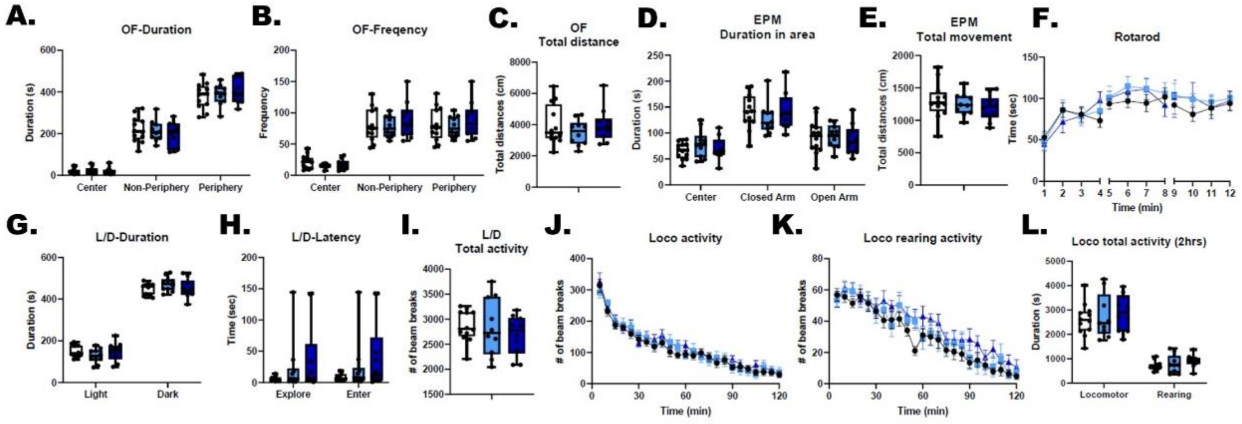

Supplemental Figure 2. Behavior testing to assess motor activity and anxiety in wild-type mice. At 3 months after vector administration, (A-C) open field (OF), (D-E) elevated plus maze (EPM), (F) rotarod, (G-I) light and dark (L/D), and (J-L) locomotor (Loco) tests were performed. Data are shown as mean ± SEM. Statistics, 2-way ANOVA. White bars, vehicle; light blue bars 1.25E+11vg dose; dark blue bars 5.00E+11vg dose; black lines, vehicle; light blue lines, 1.25E+11vg dose; dark blue lines 5.00E+11vg dose. For locomotor activity, mice were placed individually into a plastic mouse cage (18 cm x 28 cm) with minimal bedding. Each cage was placed into a dark plastic chamber. Movement was monitored by 5 photobeams in one dimension (Photobeam Activity System, San Diego Instruments, San Diego, CA) for 2 hours, with the total number of beam breaks recorded every 5 min. Mice were placed in the periphery of a novel open field environment (44 cm x 44 cm, walls 30 cm high) in a dimly lit room (approximately 60 lux) and allowed to explore for 5 min, monitored from above by a video camera connected to a computer running video tracking software (Ethovision, Noldus, Leesburg, Virginia) to determine the time, distance moved and number of entries into two areas: the periphery (5 cm from the walls) and the center (14 cm x 14cm). For elevated plus maze, mice were placed in the center of the maze (each arm 30 cm long and 5 cm wide with two opposite arms enclosed by 25 cm high walls) elevated 31 cm in a dimly lit room (approximately 60 lux) and allowed to explore for 5 min. The animals were monitored from above by a video camera connected to a computer running video tracking software (Ethovision, Noldus, Leesburg, Virginia) to determine time spent in the open arms, and the total distance moved. For rotorod motor learning, mice were placed on a stationary rotarod (Rotamex 5, Columbus Instruments, Columbus, OH). The rod was then accelerated from 4 to 40 rpm over five min. The time until each mouse fell from the rod was recorded. If a mouse held onto the rod and rotated completely around, it was treated as if it had fallen from the rod at that time. Each mouse was tested 4 times a day for 3 consecutive days with a 20-30 min intertrial interval. For dark-light activity, mice were placed into a black plexiglas chamber (25 cm x 26 cm) and allowed to explore for 2 min. After the habituation period, a small door was opened, allowing them to access the light side of the apparatus (25 cm x 26 cm, lit to approximately 1700 lux) for 10 min. The animals were monitored by 7 photobeams in the dark compartment and 8 photobeams on the light side connected to a computer which recorded the time spent in each compartment, latency to enter the light side and the number of entrances to each compartment (Med-PC IV, Med Associates, St. Albans , VT). All apparatus were wiped and allowed to dry between mice.

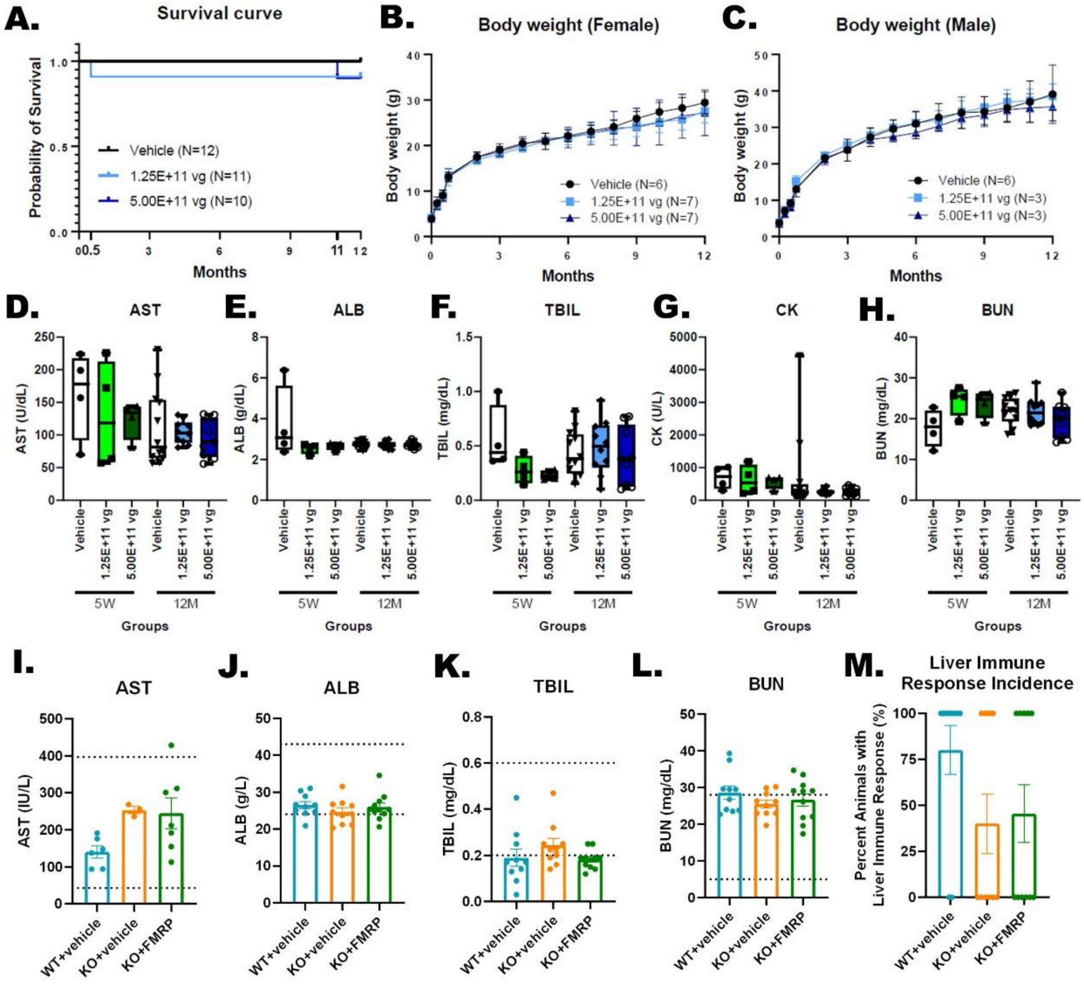

Supplemental Figure 3. Safety studies of the scAAV-JeT-hFMRPiso17 vector Mice were treated by i.t. injection at PND7-10 with a low dose (1.25E+11 vg) or high dose (5.0E+11 vg). The control group was injected with vehicle. (**A**) Survival and (**B**-**C**) average body weight of each sex are shown. Data are shown as mean ± SD. **D**–**H**. Blood chemistry tests at 5 weeks and 12 months. Aspartate aminotransferase (AST), albumin (ALB), total bilirubin (TBIL), blood urea nitrogen (BUN) and creatine kinase (CK). **I-M.** Comparison of blood and liver safety profiles from the three treatment cohorts. Mice were injected at PND2-3 with either vehicle or scAAV-JeT-hFMR1iso17 and serum and tissue samples were collected at 6 months of age. **I-L,** aspartate aminotransferase (AST, **I**), albumin (ALB, **J**), total bilirubin (TBIL, **K**), serum analyses for blood urea nitrogen (BUN, **L**). **M**. Percentage of hematoxylin and eosin-stained histological liver samples showing an immune response. There was no indication of abnormal serum levels or liver immune responses from the scAAV-JeT-hFMR1iso17 vector at 6 months post-injection. Bars = mean ± SEM; Lines = combined 95% male/female reference ranges for C57BL/6 mice 8-10 weeks (Charles River, 2012). n-values (all but AST): WT+vehicle = 10; KO+vehicle = 10; KO+FMRP = 11. n-values (AST, samples with high hemolysis excluded): WT+vehicle = 6; KO+vehicle = 3; KO+FMRP = 7.

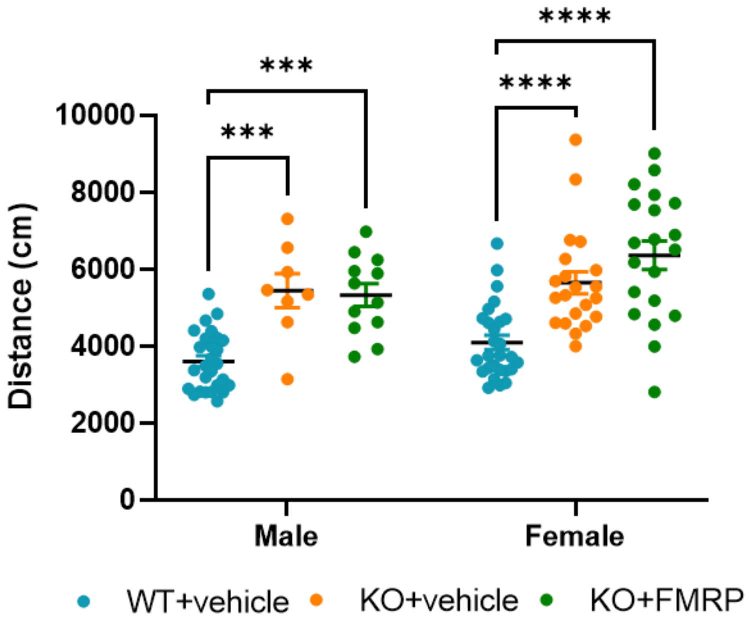

Supplemental Figure 4. Results of the open field test. Total distance travelled in 20 min. by male and female mice in the KO+vehicle and KO+FMRP groups were significantly higher compared to their respective WT+vehicle groups. * denotes statistically significant differences ($p < 0.05$) between treatment groups using post hoc Tukey's multiple comparison test. Male: WT+vehicle (n=27 males and 27 females), KO+vehicle, n=8 males and 21 females; KO+FMRP, n=12 males and 20 females.

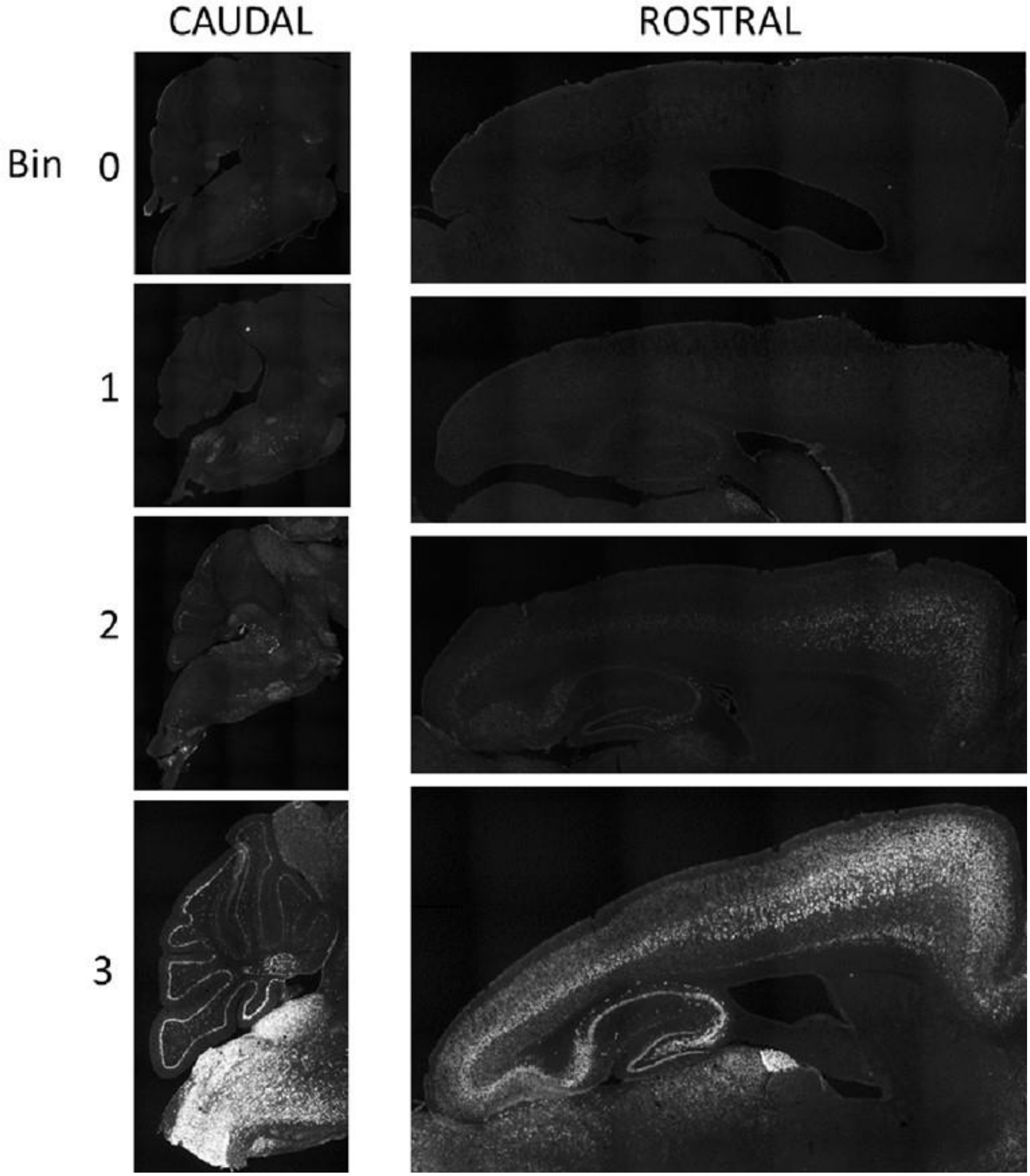

Supplemental Figure 5. Expression scoring of FMRPiso17 expression in the brain of Fmr1 KO mice. The brains of all scAAV-JeT-hFMR1iso17-injected mice that underwent physiological and/or behavioral testing were collected post-testing and stained for FMRP expression. The brains were scored according to images in the represented rubric as follows: 0 = no expression, 1 = present, but minimal expression, 2 = moderate to sub-wild type level expression, 3 = great than wild-type level expression in expressing cells. Scoring was performed independently by two observers and the scores were averaged.

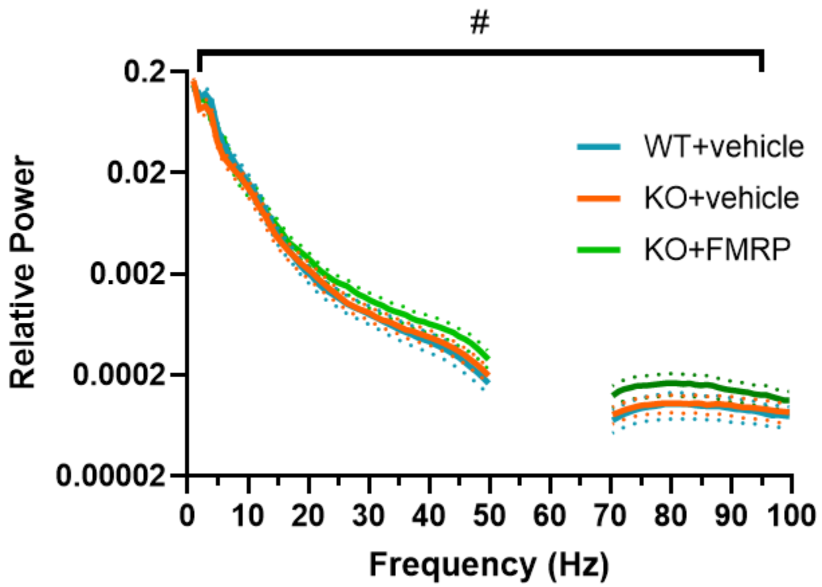

Supplemental Figure 6. Full power spectrum (0-100 Hz) comparison between WT+vehicle, KO+vehicle and KO+FMRP treatment groups. # denotes statistically significant difference by treatment using two-way ANOVA. WT+vehicle, n = 9 males; KO+vehicle, n = 16 males; KO+FMRP, n = 11 males.

A.

Certificate of Analysis Form

**Batch ID:**	NC-12-21-003
**Product Name:**	scAAV9/JeT-hsaFMR1opt-iso17-spA produced with the plasmid pSJGk- JeT-hsaFMR1opt-iso17-spA
**Manufacturing Date:**	26Jan2022
**Manufacturing Scale**	50 L
**Storage Conditions:**	≤-60˚C, Upon thawing, 4˚C
**Expiration Date**	N/A

Test	Method	Results
Endotoxin	TM005<br>LAL cartridges are a kinetic chromogenic LAL method as specified in the EP/USP	Less than 1.01 Eu/mL
Capsid Identity	TM001<br>AAV9 ELISA	4.74E+14 vp/ml
Capsid Identity	Stunner	3.86E+14 vp/mL
Titer-Vg	TM006<br>qPCR, target ITR, using an internal AAV reference standard	1.35E+14 vg/ml
Titer-Vg	TM006<br>qPCR, target FMR1, linearized plasmid used as standard	3.23E+13 vg/ml
Titer-Vg	Stunner	1.24E+14 vg/ml
Purity	TM002<br>SDS PAGE with Silver Stain [A]	VP1, VP2 and VP3 bands visible
Capsid Content	Analytical Ultra Centrifugation (AUC) [B]	34% Empty Capsids

B.

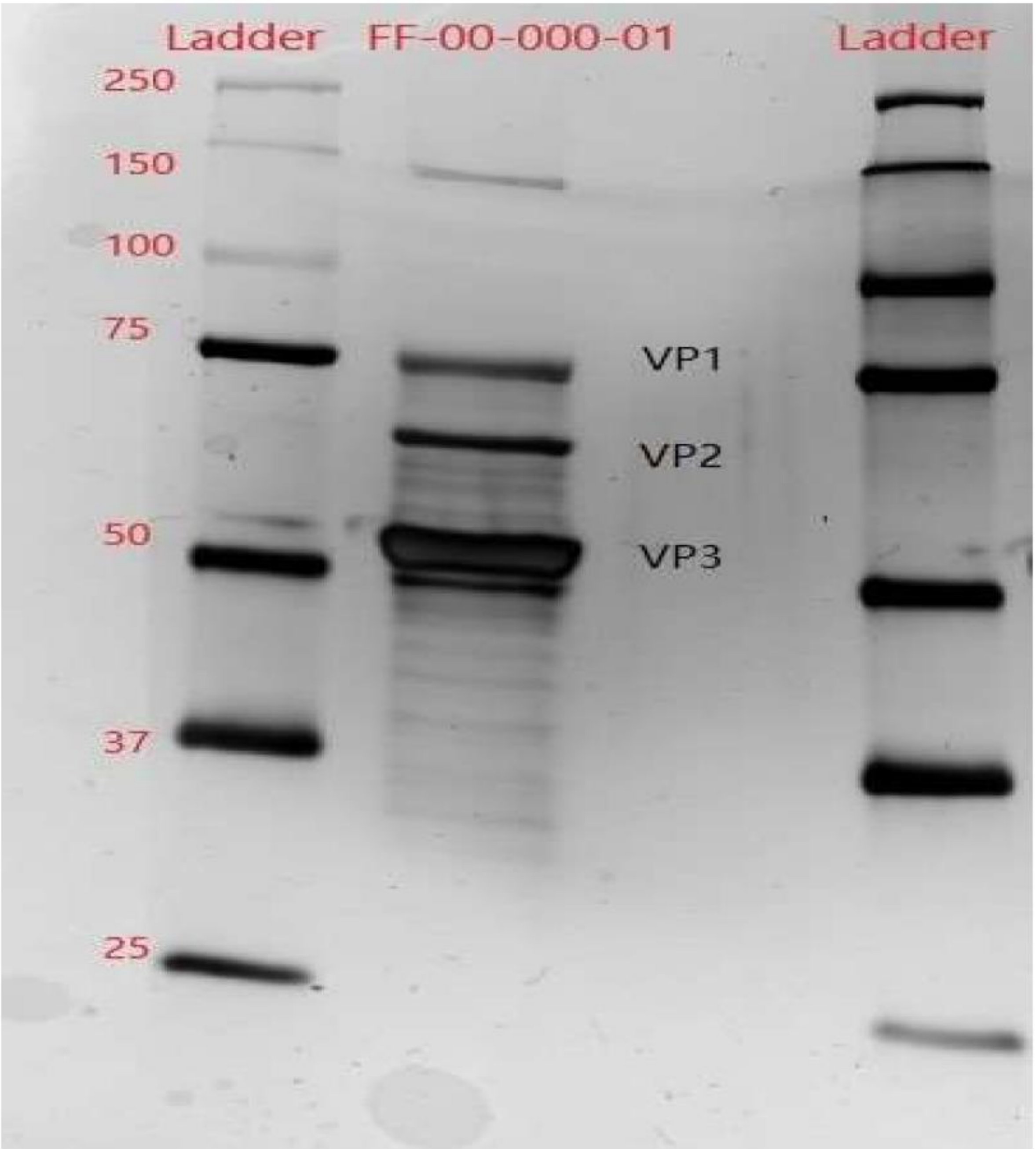

C.

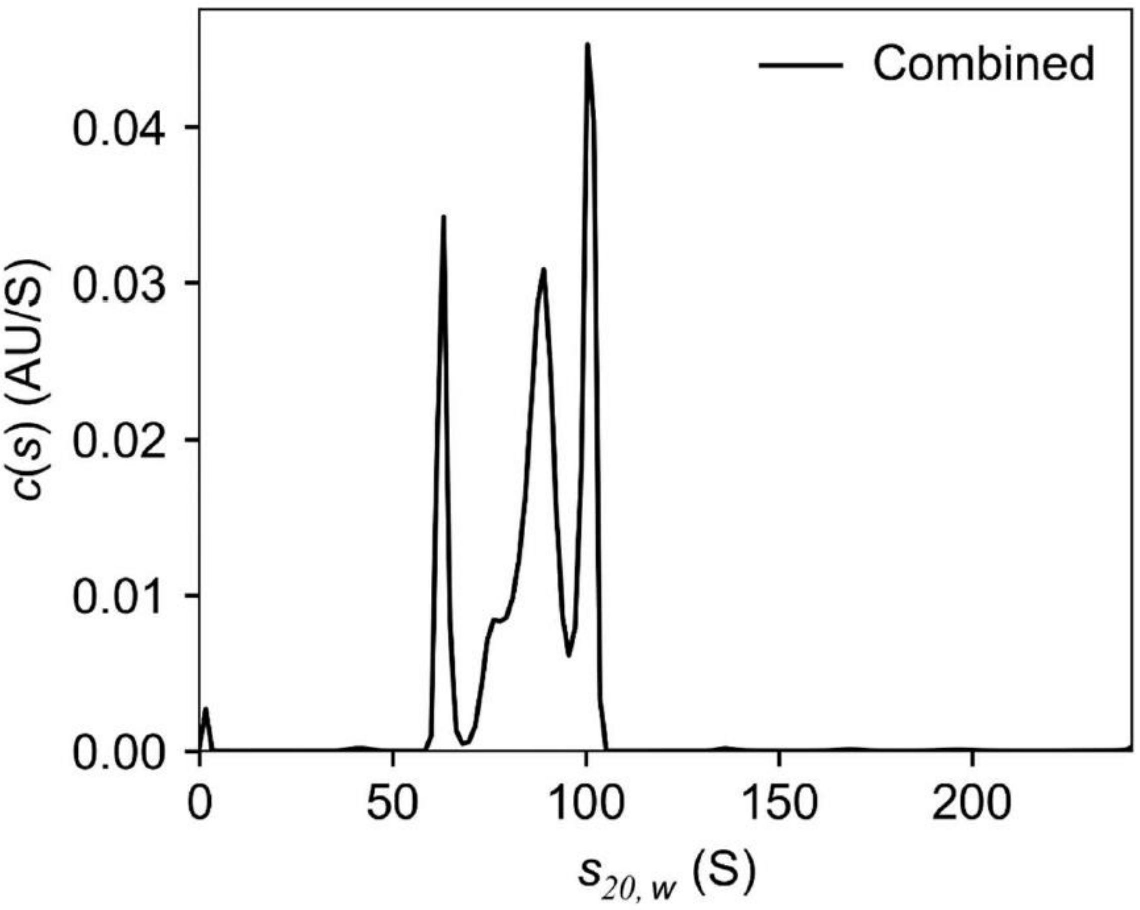

Supplemental Figure 7. Certificate of Analysis for scAAV-JeT-hFMR1iso17 vector. A. Certificate of analysis form. AAV titers determined by three different quantification methods were reported here as references: qPCR targeting the ITR, qPCR targeting FMR1 and Stunner, a method solely based on biophysical properties. We believe the titer determined by qPCR targeting FMR1 was the most accurate representation of the number of full functional capsids and hence this number was used in the manuscript. **B.** Image of SDS-PAGE silver stain gel. **C.** Graph of analytical ultra centrifugation results.

Supplemental Table 1. Component loadings of PCA.

Variables	PC1	PC2	PC3
Delta relative power	-0.1020	0.0504	-0.1275
Theta relative power	-0.5803	-0.5847	0.2559
Alpha relative power	-0.4145	-0.6746	0.4757
Beta relative power	-0.4269	-0.7504	0.4235
Gamma relative power	-0.4701	-0.6583	0.2883
% sleep time (day 1)	-0.7202	0.4092	0.2405
% sleep time (day 2)	-0.5909	0.4209	0.4377
% sleep time (day 3)	-0.7441	0.4786	0.1761
Light phase activity (day 1)	0.8190	-0.3511	0.0069
Light phase activity (day 2)	0.8444	-0.2958	0.0321
Light phase activity (day 3)	0.8408	-0.3285	0.1550
Dark phase activity (day 1)	0.5092	0.2331	0.6708
Dark phase activity (day 2)	0.4634	0.3589	0.7396
Dark phase activity (day 3)	0.5097	0.3617	0.7154
